# KITENIN promotes aerobic glycolysis through PKM2 induction by upregulating the c-Myc/hnRNPs axis in colorectal cancer

**DOI:** 10.1186/s13578-023-01089-1

**Published:** 2023-08-08

**Authors:** Mücahit Varlı, Sung Jin Kim, Myung-Giun Noh, Yoon Gyoon Kim, Hyung-Ho Ha, Kyung Keun Kim, Hangun Kim

**Affiliations:** 1 College of Pharmacy, Sunchon National University, 255 Jungang-ro, Sunchon, Jeonnam 57922 Republic of Korea; 2 Department of Pharmacology, Chonnam National University Medical School, 160 Baekseoro, Dong-gu, Gwangju, 61469 Republic of Korea; 3 Department of Pathology, Chonnam National University Medical School, 160 Baekseoro, Dong-gu, Gwanju, 61469 Republic of Korea; 4 College of Pharmacy, Dankook University, 119 Dandaero, Dongnam-gu, 31116 Cheonan-si, Republic of Korea

**Keywords:** KITENIN, ErbB4, MYO1D, Aerobic glycolysis, c-Myc, PKM2, hnRNP, Colorectal cancer, Transcriptional regulator, Disintegrator of KITENIN complex compounds

## Abstract

**Purpose:**

The oncoprotein KAI1 C-terminal interacting tetraspanin (KITENIN; vang-like 1) promotes cell metastasis, invasion, and angiogenesis, resulting in shorter survival times in cancer patients. Here, we aimed to determine the effects of KITENIN on the energy metabolism of human colorectal cancer cells.

**Experimental design:**

The effects of KITENIN on energy metabolism were evaluated using in vitro assays. The GEPIA web tool was used to extrapolate the clinical relevance of KITENIN in cancer cell metabolism. The bioavailability and effect of the disintegrator of KITENIN complex compounds were evaluated by LC–MS, in vivo animal assay.

**Results:**

KITENIN markedly upregulated the glycolytic proton efflux rate and aerobic glycolysis by increasing the expression of GLUT1, HK2, PKM2, and LDHA. β-catenin, CD44, CyclinD1 and HIF-1A, including c-Myc, were upregulated by KITENIN expression. In addition, KITENIN promoted nuclear PKM2 and PKM2-induced transactivation, which in turn, increased the expression of downstream mediators. This was found to be mediated through an effect of c-Myc on the transcription of hnRNP isoforms and a switch to the M2 isoform of pyruvate kinase, which increased aerobic glycolysis. The disintegration of KITENIN complex by silencing the KITENIN or MYO1D downregulated aerobic glycolysis. The disintegrator of KITENIN complex compound DKC1125 and its optimized form, DKC-C14S, exhibited the inhibition activity of KITENIN-mediated aerobic glycolysis in vitro and in vivo.

**Conclusions:**

The oncoprotein KITENIN induces PKM2-mediated aerobic glycolysis by upregulating the c-Myc/hnRNPs axis.

**Supplementary Information:**

The online version contains supplementary material available at 10.1186/s13578-023-01089-1.

## Background

The molecular carcinogenesis of colorectal cancer (CRC) is complex and poorly understood [1]. The pathogenesis of CRC is a multi-step process, involving both genetic and epigenetic abnormalities, which lead to the activation of oncogenes and inactivation of tumor suppressor genes in cancer cells [2].

KAI1/CD82, a transmembrane glycoprotein in the tetraspanin family, was previously reported to be a prostate-specific suppressor of metastasis. Low KAI1/CD82 activity is associated with metastasis and a poor prognosis for cancers [3–6]. A mammalian homologue of *Drosophila* VANGL1, KAI1 COOH-terminal interacting tetraspanin (KITENIN), has been shown to bind KAI1/CD82 via its COOH-terminal region [7], and to increase tumor invasiveness, cancer progression, stem cell and epithelial-mesenchymal transition (EMT) marker expression, and metastasis [8–15]. Previous studies have shown that some molecules of natural (usnic acid, atranorin, physciosporin) and synthetic (disintegrator of KITENIN complex #1125), peptides such as KITENIN dimerization-interfering peptide (KDIP), and microRNAs such as microRNA-124 origin reduce KITENIN activity [13, 16–20].

A hallmark of cancer is the reprogramming of energy metabolism [21], which includes increases in glucose uptake and utilization by cancer cells, which is known as the Warburg effect. Tumor suppressors, oncoproteins, mitochondrial DNA mutations, a hypoxic microenvironment, and other factors cause the upregulation of aerobic glycolysis in cancer [22, 23]. This alteration is mediated by activation of glucose transporters (GLUTs), hexokinase (HK), pyruvate kinase M2 (PKM2), and lactate dehydrogenase (LDH) [24]. PKM2 is highly expressed in rapidly proliferating tissues and cells, regulates aerobic glycolysis in cancer cells [25], and catalyzes the irreversible transphosphorylation between phosphoenolpyruvate (PEP) and adenosine diphosphate. Furthermore, it is the rate-limiting enzyme in glycolysis, which generates pyruvate and ATP [26].

MYO1D increases colorectal and breast cancer cell motility and viability, and to upregulated KITENIN and ErbB4 expression [27]. In a previous study, it was shown that KITENIN forms a complex with ErbB4 and promotes AP-1 signaling [12]. c-Myc is a target gene of AP-1 and induces the activity of several transporters and enzymes that are important for the glycolytic pathway, such as GLUT1, HK2, PKM2, and LDHA [28]. c-Myc also induces the transcription of hnRNPI, hnRNPA1, and hnRNPA2, resulting in preferential expression of the PKM2 isoform [29]. High levels of PKM2 expression cause greater nuclear translocation of PKM2 and positive feedback on the transcriptional activity of the β-catenin, c-Myc, and HIF-1α genes [30]. In addition, HIF-1α expression promotes the activity of the glycolytic pathway through the upregulation of GLUT1, HK1, HK2, and LDHA [31–33].

The expression and biological role of KITENIN in malignant tumors have not yet been characterized, nor has the relationship of KITENIN expression with the metabolism of CRC cells. Therefore, in the present study, we aimed to address these deficiencies, and show that the expression of KITENIN is closely related to aerobic glycolysis and its transcriptional regulators in CRC. These findings suggest that KITENIN represents a potential target for the development of novel cancer cell metabolism-based therapies.

## Material and method

### Cell culture

Human colorectal cancer cell CaCo2, HCT116 and murine CT26 cell were used. Cell line modified to Empty vector or KITENIN overexpression cells. All cells were cultured in DMEM (GenDepot, Katy, TX, USA) supplemented with 10% fetal bovine serum (FBS) and 1% penicillin–streptomycin solution. 5% CO2 in a humidified atmosphere at 37 °C was used for incubation of the cells. A hypoxic state was created by incubation with 300 µM Cobalt (II) chloride (CoCl2).

### Invasion assay

Cell invasion was assessed using Boyden chambers (Corning, New York, NY, USA) coated with 1% gelatin. CRC cells in 100 µl DMEM containing 0.2% bovine serum albumin (BSA) were plated onto precoated inserts and incubated for 24 h. The lower chamber was filled with 600 µl DMEM containing 0.2% BSA and 10 µg/ml fibronectin (EMD Millipore Corp., Billerica, MA, USA) as a chemoattractant. After incubation, cells in the upper chamber were fixed and stained using a Diff-Quick kit (Sysmex, Kobe, Japan). Observed using a Nikon Eclipse 400 fluorescence microscope (Nikon Intech, Co., Ltd.). Invaded cells were counted in five random fields of each Boyden chamber and analyzed by iSolution FLAutosoftware.

### siRNA and plasmid transfection

siRNA interference experiments, we purchased from siRNA specific to human MYO1D (Thermo Scientific and Invitrogen), purchased from siRNA specific to human KITENIN (Thermo Scientific and Invitrogen), and siPKM2 specific to human (CCA UAA UCG UCC UCA CCA A) (Bioneer). The si-RNA duplexes were prepared and transfected according to a protocol provided by Lipofectamine^™^ RNAiMAX. Cells were treated for 24 h or 48 h to allow for maximum knockdown, followed by qRT-PCR, Western blot analysis, invasion assays and Seahorse XF instrument analysis.

PKM (Myc-DDK-tagged)-Human pyruvate kinase, muscle (PKM2), transcript variant 2 purchased from Origene. The plasmid was prepared and transfected according to a protocol provided by Lipofectamine^™^ LTX with Plus Reagent.

### Extracellular acidification rate (ECAR) measurements

To measure real-time changes in ECAR, an XF96 extracellular flux analyzer (Agilent, Santa Clara, CA, USA) was used. First, CRC cells were seeded at 5 × 10^3^ cells/well, incubated overnight in culture medium (2% FBS), and then incubation for 48 h. On the day of analysis, the plated cells were washed twice and loaded (180 μL) with assay medium enriched with glucose, sodium pyruvate, and glutamine. While incubating the cells for 1 h at 37 °C in a non-CO2 incubator prior to analysis, 0.5 μM rotenone (Rot) + antimycin A (AA) and 50 mM 2-deoxy-d-glucose (2-DG) were loaded into the hydrated sensor cartridge for ECAR analysis (glycolytic rate assay) [34]. ECAR was measured on Seahorse XF instrument (Agilent). The results were analyzed using the Wave software (Agilent).

### Oxygen consumption rate (OCR) measurements

To measure real-time changes in OCR, an XF96 extracellular flux analyzer (Agilent, Santa Clara, CA, USA) was used. First, CRC cells were seeded at 5 × 10^3^ cells/well, incubated overnight in culture medium (2% FBS), and then incubation for 48 h. 1 μM oligomycin, 1 μM carbonyl cyanide 4- (trifluoromethoxy) phenylhydrazone (FCCP), and 0.5 μM Rotenone (Rot) + antimycin A (AA) were loaded for OCR analysis (Cell Mito Stress Test) [34]. OCR measured on a Seahorse XF instrument (Agilent). The results were analyzed using the Wave software (Agilent).

### Cell cycle arrest assay

CaCo2/EV and CaCo2/KITENIN cells were seeded in six-well plates at a density of 2 × 10^5^ cells/well, cultured overnight, after that transfection si-MYO1D for 24, trypsinized, and washed with FACS wash solution. First trypsin solution was added and incubated for 10 min and then RNase inhibitor was added and incubated for 10 min at room temperature. Next, the samples were centrifuged, the supernatants were removed, and the pellets were resuspended in 100 µL of 4 mg/mL PI (Sigma-Aldrich, St. Louis, MO, USA) and incubated for 2 h in the dark at 4 °C. Flow cytometry was performed with a CytoFLEX instrument (Beckman Coulter Life Sciences, Indianapolis, IN, USA).

### Quantitative real-time polymerase chain reaction (PCR)

Total RNA was extracted from CRC cells using RNAiso Plus (TaKaRa, Otsu, Japan). cDNA was synthesized from one microgram of RNA by M-MLV (Invitrogen, Carlsbad, CA, USA). SYBR Green reagents (Enzynomics, Seoul, Korea) were used to determine relative expression of candidate genes. The list of primers used in this research is reported in Additional file 1: Table S1. CFX instrument (Bio-Rad, Hercules, CA, USA) was used to perform the analysis.

### Correlation analysis and survival analysis

GEPIA (Gene Expression Profiling Interactive Analysis, RRID:SCR_018294, http://gepia.cancer-pku.cn/) (http://gepia.cancer-pku.cn/) was extracted to estimate the differential expression level between VANGL1 and metabolic and transcription regulators in colon samples [35]. The Pearson Coefficient was used to analyse the expression correlation between the two genes. The log2 of the transcript per million is known as log2(TPM). Hazard ratio (HR) and survival curve for the indicated gene and ESCC cohort were determined by log-rank test and Kaplan–Meier method on pan-cancer. The p value < 0.05 was noted statistically significant.

Disease Specific cumulative survival data with high expression of KITENIN and low vs high expression of PKM2 were generated from Chonnam National University Hwasun Hospital patient records. The present study was approved by the Institutional Review Board of Chonnam National University Hwasun Hospital.

### Western blotting

Whole cell lysates were prepared as follow: cells were plated into 6-well plates, incubated for overnight. After indicated incubation, cells were washed twice with ice-cold PBS, and lysed by lysis buffer. Concentrations of protein in lysate were determined by BCA assay (Thermo Fisher Scientific, Waltham, MA USA). Antibodies (Additional file 1: Table S2) were detected by horseradish peroxidase-conjugated secondary antibody (Thermo Fisher Scientific) using the Immobilon Western Chemiluminescent HRP Substrate Kit (Millipore, Billerica, MA, USA) and luminescence imaging. Relative density of bands was obtained by normalizing the density of α-tubulin, β-actin or GAPDH bands in each sample using Multi-Gauge 3.0. Values were presented as arbitrary units of densitometry of corresponding to signal intensity.

### Immunoprecipitation

Immunoprecipitation was performed as previously described [36]. Immunoprecipitation was performed using lysates from CaCo2/EV and CaCo2/KITENIN cells that were incubated with primary antibody for overnight at 4 °C and pulled down with Protein A/G Sepharose (Thermo Scientific, catalog number: 20422) for 3 h. The immunoprecipitated proteins were washed 6 time with the same buffer, and bound proteins were resolved by SDS-PAGE followed by immunoblotting.

### Subcellular fractionation

Cytoplasmic and nucleus fractions were prepared by a subcellular protein fractionation protocol according to the Thermo Scientific instructions (NE-PER^™^ Nuclear and Cytoplasmic Extraction Reagents, catalog number: 78833). Fraction purity was assessed by probing for tubulin or β-actin for the cytoplasm and Lamin B for the nucleus.

### Optimization of DKC1125

With the goal of developing a potent and solubility, inhibiting the KITENIN-induced upregulation of aerobic glycolysis and tumorigenesis as compared to DKC1125, an analysis of the binding mode of DKC1125 was initiated. As part of the lead optimization process, the initial derivatization of DKC1125 began with the introduction of various substituents at the phenyl position in the phenethyl amide core. These results that the structural modification of DKC1225 at the *meta*-phenyl position followed by the dimethylamino group led to the discovery of DKC-C14S with increased solubility and activity dramatically.

### In vivo* pharmacokinetic study*

Eight-week-old adult male ICR mice and SD rats were used in the present study. The animals were housed four per cage and kept in a vivarium maintained the temperature at 23 ± 2 °C and the humidity at 50 ± 10% with a 12 h: 12 h alternating light/dark cycle. The animals were fasted for at least 12 h prior to the start of the experiments and freely supplied water. On the morning of the test, the animals were anaesthetized with a mixture of alfaxan and rompun (75:25, *v/v*). For oral administration studies, the carotid artery was cannulated using a polyethylene tube for efficient blood sampling. For intravenous administration studies, the carotid artery and jugular veins were cannulated for blood sampling and drug administration, respectively. Each cannula was exteriorized to the dorsal side of the neck. Then, each animal was individually housed in a metabolism cage and allowed to move freely. A recovery time of 3–4 h from anesthesia was allowed before drug administration. For oral administration, DKC-C14S (4 mL/kg dissolved in 50% PEG400 water solution) solution was administered using a gastric gavage tube at doses of 10 mg/kg. For intravenous administration, the same solution was administered via jugular vein over 1 min at doses of 2 mg/kg. Animal blood samples were collected using automated blood sampling system (Culex, BASi, West Lafayette, IN, USA) and the time point were as follows: 5, 15, 30, 60, 120, 240 and 360 min after administration. The blood samples were immediately centrifuged at 12,000 rpm for 5 min. Each plasma sample was transferred into a new microtube and stored at − 80 °C until used for LC–MS/MS analysis. Sample analysis was conducted by validated LC–MS/MS method. The liquid chromatographic system was Ultimate^®^ 3000 HPLC unit (Dionex, Sunnyvale, CA, USA) which connected to AB SCIEX API 3200 triple quadrupole mass spectrometer (Applied Biosystems Sciex, Toronto, Ontario, Canada) with an electrospray ionization (ESI). All experimental procedures were approved by Dankook University’s Institutional Animal Care and Use Committee.

### In vivo* tumor growth*

Five- to six-week-old BALB/c mice were obtained from OrientBio (Seongnam, Korea) and housed in metal cages with free access to water and food. In brief, CT-26/KITENIN-iRFP-expressing cells (2 × 10^5^ cells/mouse) were injected subcutaneously into the right flank of each mouse. After 1 week later, mice were intraperitoneally injected with vehicle or DKC-C14S at a dose of 5 mpk once every 2 days. Using a fluorescence-labeled organism bioimaging instrument (FOBI) technology, near-infrared fluorescence bioimaging was promptly achieved (Cellgentek, Osong, Korea). After sacrifice, mouse tumor tissues could be properly isolated, and each sample's tumor weight was calculated. The Chonnam National University Medical School Research Institutional Animal Care Committee provided oversight for all animal trials, and the committee also approved all experimental procedures (CNU IACUC-H-2020-12).

### Statistical analysis

Data represents as means ± standard deviation. Statistical analyses were carried out using the Sigma Plot 12.5 software (RRID:SCR_003210, Systat Software, Erkrath, Germany). The statistically significant between two groups was compared using the student’s t test. Unless indicated otherwise, a p-value ≤ 0.05 was considered significant.

## Results

### Overexpression of KITENIN upregulates aerobic glycolysis

**Fig. 1:**
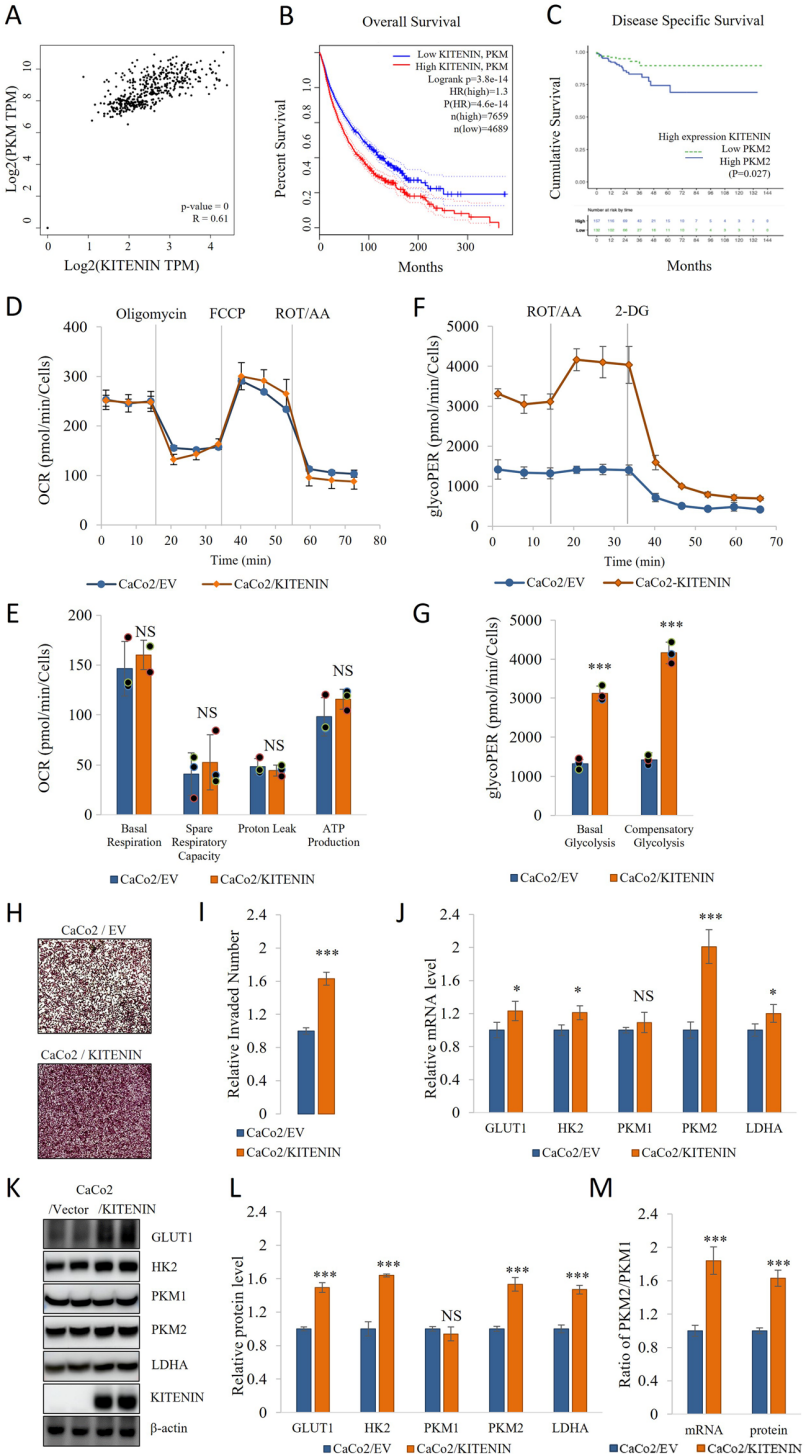
Overexpression of KITENIN causes an induction of aerobic glycolysis but no change in mitochondrial respiration. **A** The GEPIA web tool was used to analyze the relationship between the expression of KITENIN and that of PKM mRNA levels in COAD samples. **B** Overall survival curve of between KITENIN-PKM, according to the GEPIA database. **C** Disease-specific survival curve of between among the KITENIN high expression and high vs low PKM2 patients. **D**–**E** Oxygen consumption rate (OCR) was measured using a Cell Mito Stress Test Kit on a Seahorse XF96 extracellular flux analyzer. OCR was measured, and then oligomycin (1 μM), FCCP (0.5 μM), rotenone (1 μM), and antimycin A (1 μM) were added sequentially to assess basal respiration, spare respiratory capacity, proton leak, and ATP production, respectively. **F**–**G** CaCo2/EV and CaCo2/KITENIN cells were incubated for 48 h and then their metabolism was studied. The extracellular acidification rate (ECAR) and oxygen consumption rate (OCR) were assessed using a Glycolytic Rate Assay Kit on a Seahorse XF96 extracellular flux analyzer. The assay utilizes both ECAR and OCR measurements to determine the glycolytic proton efflux rate (glycoPER). GlycoPER was measured at two time points, and then rotenone (1 μM)/antimycin A (1 μM) and 2-DG (50 mM) were added in sequence. **H**–**I** Transwell invasion assays were performed using CaCo2/EV and CaCo2/KITENIN cells. Representative images and the relative number of invaded cells are shown. **J** To elucidate the mechanism by which KITENIN affects glycolysis, the relative mRNA expression of molecules mediating the Warburg effect (GLUT1, HK2, PKM1, PKM2, and LDHA) was measured in CaCo2/EV and CaCo2/KITENIN cells incubated for 48 h. **K**–**L** Protein expression of GLUT1, HK2, PKM1, PKM2, and LDHA. α-Tubulin or actin served as the loading control. **M** The PKM2/PKM1 ratio was calculated using the relative mRNA expression and protein level of PKM1 and PKM2 after 48 h. Data are the mean ± standard deviation. * *p* < 0.05; ** *p* < 0.01; *** *p* < 0.001

KITENIN is an oncogene that is overexpressed in many different types of cancer, including CRC. Using the GEPIA web tool, we identified that KITENIN and several transcriptional regulators related the aerobic glycolysis are overexpressed in colon cancer tumors compared with normal tissue (Additional file 1: Fig S1). Even in the presence of oxygen and functioning mitochondria, aerobic glycolysis is a crucial bioenergetic process in cancer cells (the Warburg effect) [37]. The GEPIA web tool was used to identify the survival rate and correlation between the expression of KITENIN-PKM2, KITENIN-GLUT1, KITENIN-HK2, and KITENIN-LDHA, KITENIN-MCT1 levels in samples (Fig. 1A, B, Additional file 1: Fig S2). Disease-specific survival was found to be better of the KITENIN and high expression of PKM2 (blue, n = 157) *vs*. low expression of PKM2 (green, n = 132) level in patients (Fig. 1C). The results showed that KITENIN correlated with metabolic markers and their co-expression caused low survival. We assessed the glycolytic activity of Caco2/EV and CaCo2/KITENIN cells by measuring the rate of extracellular acidification (ECAR) and OCR using a Seahorse XF Glycolytic Rate Assay, an antimycin/rotenone mixture, and 2-deoxy-D-glucose (2-DG) in order to calculate the glycolytic proton flux (glycoPER). Mitochondria are the key site for cellular energy production, and therefore the targeting of mitochondrial respiration is considered to represent a valuable strategy for cancer therapy [38]. However, KITENIN overexpression did not affect these mitochondrial pathways. Mitochondrial respiration was assessed by the real-time tracking of oxidative phosphorylation, expressed as OCR, and the sequential addition of oligomycin, FCCP, and antimycin/rotenone permit the assessment of mitochondrial function, including basal respiration, maximal respiration, ATP production, proton leak, and spare respiratory capacity [39]. As shown in Fig. 1D, E, the overexpression of KITENIN did not induce differences in mitochondrial respiration from that of cells transfected with empty vector. However, as shown in Fig. 1F, G and Additional file 1: Fig S3B, glycolytic parameters, including the basal and compensatory glycolytic activities, were significantly induced by the overexpression of KITENIN.

Hyperactivation of the glycolytic pathway increases cellular acidity, invasion, and angiogenesis [40], and we found that the overexpression of KITENIN induces cell invasion (Fig. 1H, I). To determine how KITENIN overexpression induces the Warburg effect, we measured the expression of several key transporters and enzymes involved in the Warburg effect: GLUT-1, HK2, PKM2, and LDHA (Fig. 1J–L, Additional file 1: Fig S3C–D, S4). Another isoform of PKM, PKM1, regulates glycolysis, and if the PKM1/PKM2 ratio is high, glycolysis is inhibited and the cell switches to oxidative phosphorylation [41]. We found that KITENIN overexpression is associated with a high PKM2/PKM1 ratio (Fig. 1M).

### Knockdown of MYO1D inhibits KITENIN and cell cycle progression, and low KITENIN/ErbB4 expression reduces aerobic glycolysis

**Fig. 2:**
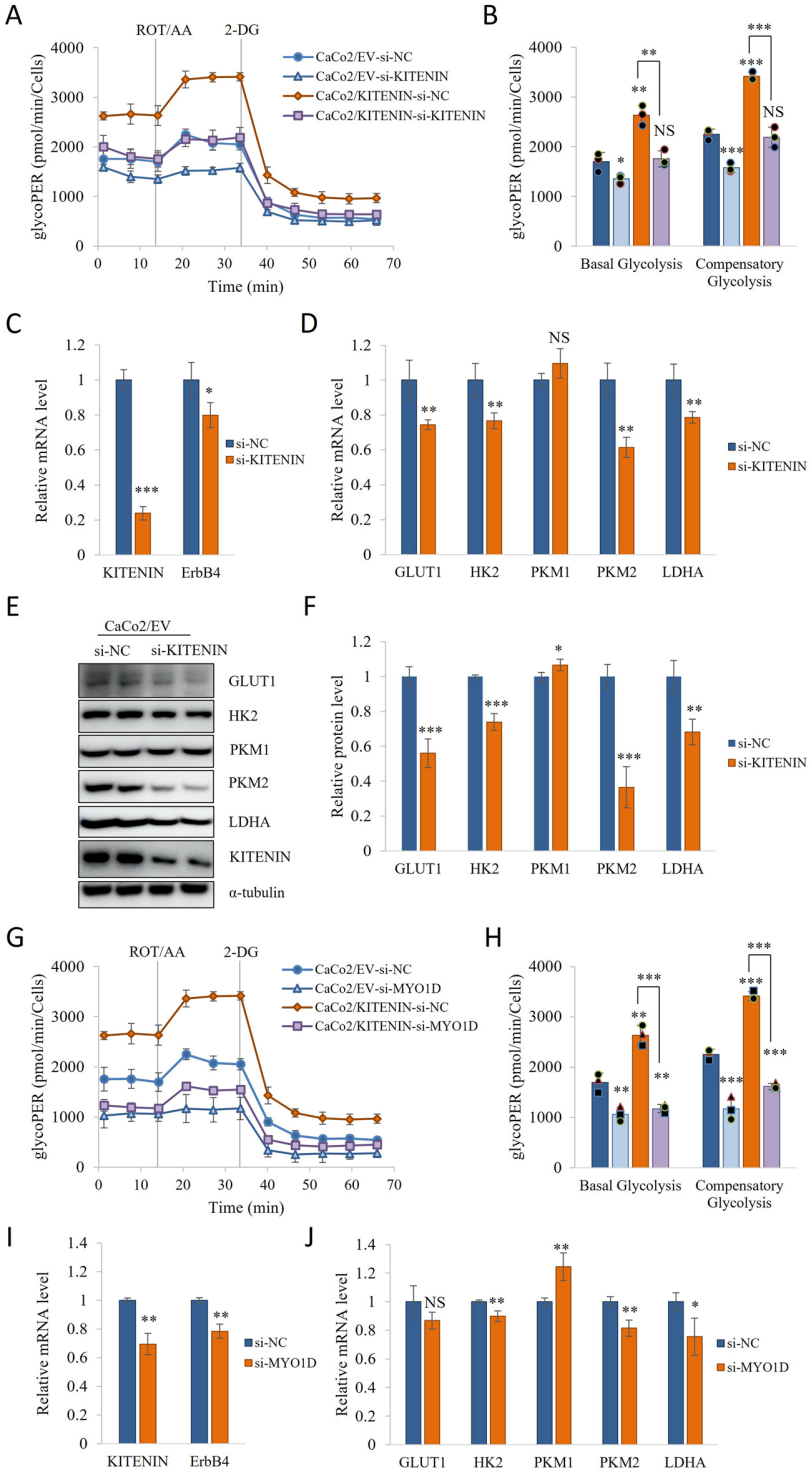
KITENIN knockdown reduces the expression of molecules responsible for the Warburg effect. **A**–**B** CaCo2/EV and CaCo2/KITENIN cells were transfected with si-KITENIN and then their metabolism was assessed. The extracellular acidification rate (ECAR) and oxygen consumption rate (OCR) were measured using a Glycolytic Rate Assay Kit on a Seahorse XF96 extracellular flux analyzer. To measure glycolytic rate, the assay utilizes both ECAR and OCR measurements to determine the glycolytic proton efflux rate (glycoPER). GlycoPER was measured at two time points, and then following the sequential injection of rotenone (1 μM)/antimycin A (1 μM) and 2-DG (50 mM). **C** Effect of si-KITENIN on the mRNA expression of KITENIN and ErbB4. **D**–**F** Relative mRNA expression and protein level of molecules mediating the Warburg effect (GLUT1, HK2, PKM1, PKM2, and LDHA) in CaCo2/EV cells transfected si-negative control or si-KITENIN for 48 h. **G**–**H** CaCo2/EV and CaCo2/KITENIN were transfected with si-MYO1D and then their metabolism was assessed. The extracellular acidification rate (ECAR) and oxygen consumption rate (OCR) were measured using a Glycolytic Rate Assay Kit on a Seahorse XF96 extracellular flux analyzer. To assess the glycolytic rate, the assay utilizes both ECAR and OCR measurements to determine the glycolytic proton efflux rate (glycoPER). GlycoPER was measured at two time points, and then following the sequential injection of rotenone (1 μM)/antimycin A (1 μM) and 2-DG (50 mM). **I**–**J** Relative mRNA expression of KITENIN, ErbB4, GLUT1, HK2, PKM1, PKM2, and LDHA in CaCo2/EV cells for incubated 48 h after transfection with si-MYO1D. Data are mean ± standard deviation. * *p* < 0.05; ** *p* < 0.01; *** *p* < 0.001, NS, no significant difference between groups

Receptor tyrosine kinases (RTKs) are involved in numerous biological processes, including cell survival, differentiation, and growth, and have been targeted for a number of cancer therapies [42–44]. Previous studies have shown that EGFR is co-expressed with ErbB4 and KITENIN [8, 9, 12, 14], and that EGFR signaling upregulates glycolysis and PKM2 expression [45]. MYO1D binds to the kinase domains of members of the EGFR family on plasma membranes, which causes their activation and contributes to carcinogenesis. We have previously shown that the knockdown of MYO1D reduces the protein levels of KITENIN and ErbB4 [27]. In the present study, we determined the effects of silencing the plasma membrane protein MYO1D and KITENIN on glycolysis and found that the knockdown of KITENIN and MYO1D reduced the glycoPER (Fig. 2A, B, G, H, Additional file 1: Fig S5A, B). Subsequently, to determine the effects of KITENIN knockdown on key mediators of glycolysis, we performed qRT-PCR and Western blotting. The knockdown of KITENIN and MYO1D reduced ErbB4, GLUT1, HK2, PKM2, and LDHA mRNA expression (Fig. 2C, D, I, J, Additional file 1: Fig S5C–D), and KITENIN knockdown reduced the GLUT1, HK2, PKM2, and LDHA protein levels (Fig. 2E, F, Additional file 1: Fig S5E, F). Also, we showed MYO1D knockdown decreases the protein level of KITENIN, GLUT1, HK2, PKM2 and LDHA level in Caco2/EV cells (Additional file 1: Fig S6A–D).

We next assessed the effects of MYO1D knockdown on the cell cycle in KITENIN-overexpressing cells, by comparison to cells transfected with empty vector, and found that MYO1D knockdown caused cell cycle arrest. Specifically, MYO1D knockdown in CaCo2/EV and CaCo2/KITENIN-overexpressing cells accumulated the G1 and S phases. Therefore, MYO1D knockdown caused the population to decline in the G2/M phase (Additional file 1: Fig S7).

### PKM2 has a role in KITENIN overexpression

**Fig. 3:**
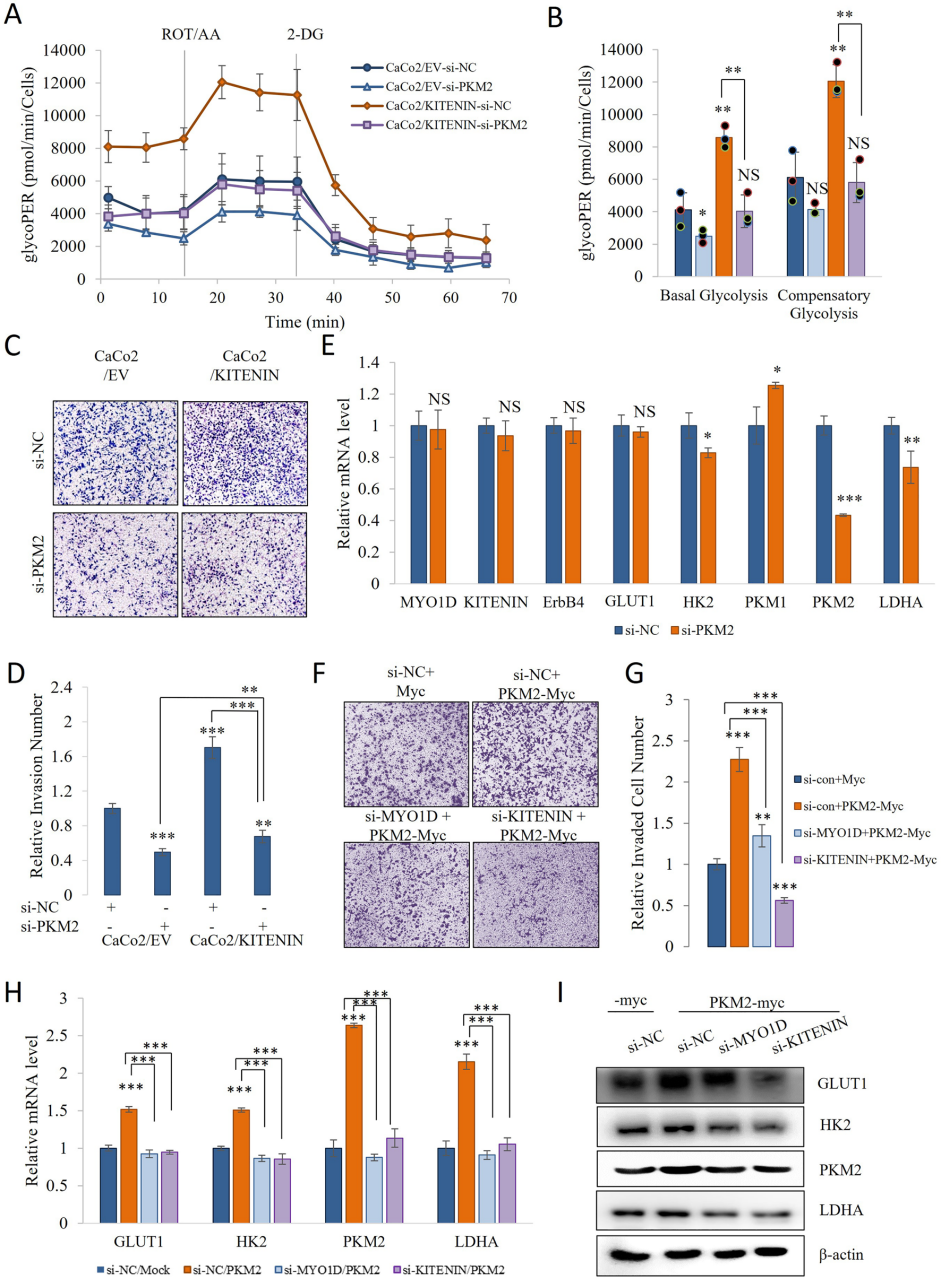
Relationship between PKM2 and KITENIN. **A**–**B** CaCo2/EV and CaCo2/KITENIN cells were transfected with si-PKM2 and then their metabolism was analyzed. The extracellular acidification rate (ECAR) and oxygen consumption rate (OCR) were measured using a Glycolytic Rate Assay Kit on a Seahorse XF96 extracellular flux analyzer. To assess the glycolytic rate, the assay utilizes both ECAR and OCR measurements to determine the glycolytic proton efflux rate (glycoPER). GlycoPER was measured at two time points, and then following the sequential injection of rotenone (1 μM)/antimycin A (1 μM) and 2-DG (50 mM). **C**–**D** Invasion assay for CaCo/EV and CaCo2/KITENIN cells transfected with si-negative control or si-PKM2, using fibronectin as a chemoattractant. KITENIN-overexpressing cells showed greater invasiveness than empty vector-transfected cells. The stained invading cells were counted and the numbers in each group are shown in a bar graph. **E** Relative mRNA expression of MYO1D, KITENIN, ErbB4, GLUT1, HK2, PKM1, PKM2, and LDHA in CaCo2/EV transfected with si-PKM2. **F**–**G** Knockdown of KITENIN and MYO1D suppressed enhanced invasion capacity by PKM2. CaCo2 cells were transfected with si-KITENIN and si-MYO1D for 24 h followed by transfection of the plasmid, a construct expressing myc-tagged PKM2 (PKM2-myc) for 24 h and subjected to the transwell invasion assay. The stained invading cells were counted and the numbers in each group are shown in a bar graph. **H**–**I** Knockdown of KITENIN and MYO1D suppressed induced GLUT1, HK2, PKM2 and LDHA mRNA and protein levels by PKM2. Data are the mean ± standard deviation, n = 3 * *p* < 0.05; ** *p* < 0.01; *** *p* < 0.001, NS, no significant difference between groups

Depending on the expression of KITENIN, the expression of PKM2 varies significantly. Therefore, to evaluate the function of PKM2 in KITENIN-overexpressing CaCo2 cells, we transfected a si-PKM2 or si-negative control. PKM2 knockdown significantly reduced glycolytic proton efflux (glycoPER) under KITENIN overexpression condition (Fig. 3A, B). Next, we determined how cell motility, which is affected by glycolysis-derived lactic acid accumulation, was affected by si-PKM2 transfection under both conditions, and obtained results that were consistent with those of the glycoPER results. KITENIN-overexpressing cells show significantly greater invasion, migration, and proliferation [46], and we found that the knockdown of PKM2 significantly reduced invasion under both conditions (Fig. 3C, D).

To determine whether si-PKM2 specifically reduces PKM2 expression, we performed Western blotting to measure the protein expression of PKM2, PKM1, and KITENIN in CaCo2/EV and CaCo2/KITENIN cells (Additional file 1: Fig S8A, B); and qRT-PCR to measure the mRNA expression of KITENIN, ErbB4, and the mediators of the Warburg effect GLUT1, HK2, PKM2, and LDHA (Fig. 3E). Furthermore, we investigated whether the knockdown of KITENIN and MYO1D suppressed PKM2-mediated induction of cell invasion and aerobic glycolysis markers. PKM2-myc transfection statistically significantly induced cell invasion relative to myc-transfected (mock transfection) in CaCo2/EV cells, while si-KITENIN and si-MYO1D transfection significantly suppressed PKM2-mediated cell invasion (Fig. 3F, G). PKM2 transfection activated the aerobic glycolysis markers GLUT1, HK2, and LDHA. The knockdown of KITENIN and MYO1D on the activated markers significantly suppressed PKM2-mediated induction of GLUT1, HK2, PKM2 and LDHA mRNA expressions and protein levels (Fig. 3H, I).

### KITENIN increases the expression of the AP-1 target gene c-Myc, and c-Myc increases PKM2 activity through the upregulation of hnRNP isoforms

**Fig. 4:**
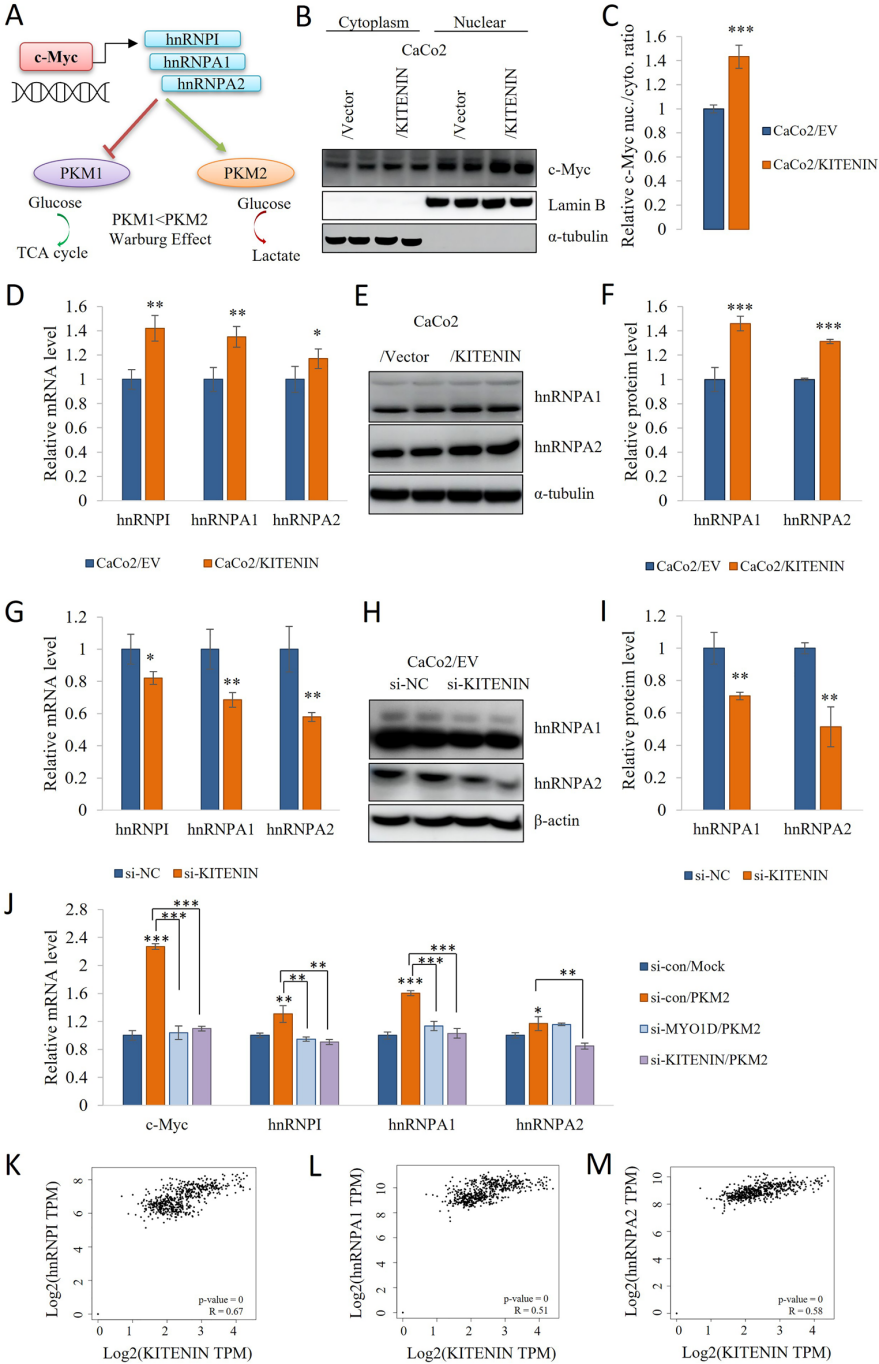
c-Myc promotes PKM2 expression via hnRNPs isoforms. **A** Schematic illustration of how c-Myc increases PKM2 expression via hnRNPs isoforms. **B**–**C** Western blot analysis of the cytoplasmic and nuclear expression of c-Myc in CaCo2/EV and CaCo2/KITENIN cells. **D** hnRNPI (PTBP1), hnRNPA1, and hnRNPA2 mRNA expression in CaCo2/EV and CaCo2/KITENIN cells. **E**–**F** hnRNPA1 and hnRNPA2 protein level in CaCo2/EV and CaCo2/KITENIN cells. **G** hnRNPI (PTBP1), hnRNPA1, and hnRNPA2 mRNA expression in KITENIN-silenced cells. **H**–**I** KITENIN knockdown reduces the protein level of hnRNPA1 and hnRNPA2 in CaCo2/EV cells. **J** Knockdown of KITENIN and MYO1D inhibit c-Myc and hnRNPI, hnRNPA1, hnRNPA2 mRNA levels on PKM2-myc transfected cells. CaCo2 cells were transfected with si-KITENIN and si-MYO1D for 24 h followed by transfection of the plasmid, a construct expressing myc-tagged PKM2 (PKM2-myc) for 24 h and subjected to the qRT-PCR. Data are mean ± standard deviation, n = 3. * *p* < 0.05; ** *p* < 0.01; *** *p* < 0.001, NS, no significant difference between groups. (**K–M)** The GEPIA web tool was used to analyze the relationships of the mRNA expression of KITENIN with that of hnRNPI (PTBP1), hnRNPA1, and hnRNPA2 in COAD samples

c-Myc is a key regulator in cancer cells, and its overexpression promotes metabolic changes and cell proliferation [47, 48]. Therefore, we compared the c-Myc levels in nuclear and cytoplasm extracts of Caco2/EV and CaCo2/KITENIN cells and found that the overexpression of KITENIN increased nuclear c-Myc expression (Fig. 4B, C). We analyzed protein interactions by immunoprecipitation (IP) with the c-Myc antibody using CaCo2/EV and CaCo2/KITENIN cells. Our results show that c-Myc-PKM2 interaction is increased in KITENIN overexpressed conditions (Additional file 1: Fig S9). c-Myc upregulates the transcription of hnRNPI (PTBP1), hnRNPA1, and hnRNPA2, and the upregulation of hnRNP isoforms by c-Myc increases the PKM2 (exon 10)/PKM1 (exon 9) ratio [29, 49, 50] (Fig. 4A). We next measured the hnRNPI (PTBP1), hnRNPA1, and hnRNPA2 mRNA expressions and the hnRNPA1 and hnRNPA2 protein levels in KITENIN-overexpressing and silenced cells (Fig. 4D–I). Also MYO1D knockdown reduce the protein level of c-Myc, hnRNPA1 and hnRNPA2 (Additional file 1: Fig S6E, F). Next, we investigated how the levels of c-Myc and hnRNPs induced by PKM2 were affected by the silencing of KITENIN and MYO1D. Our results showed that silencing KITENIN and MYO1D significantly suppressed c-Myc and hnRNPI, hnRNPA1 and hnRNPA2 levels compared to PKM2-myc and control si-RNA transfected cells (Fig. 4J and Additional file 1: Fig S10). The expression of KITENIN was associated with the expression of hnRNPI, hnRNPA1, and hnRNPA2 in colon cancer using the GEPIA analysis tool (Fig. 4K–M). Thus, c-Myc promotes PKM2 expression by upregulating hnRNP isoforms.

### KITENIN increases the transcriptional regulators involved in aerobic glycolysis, and promotes the nuclear PKM2, β-catenin, and HIF-1α

**Fig. 5:**
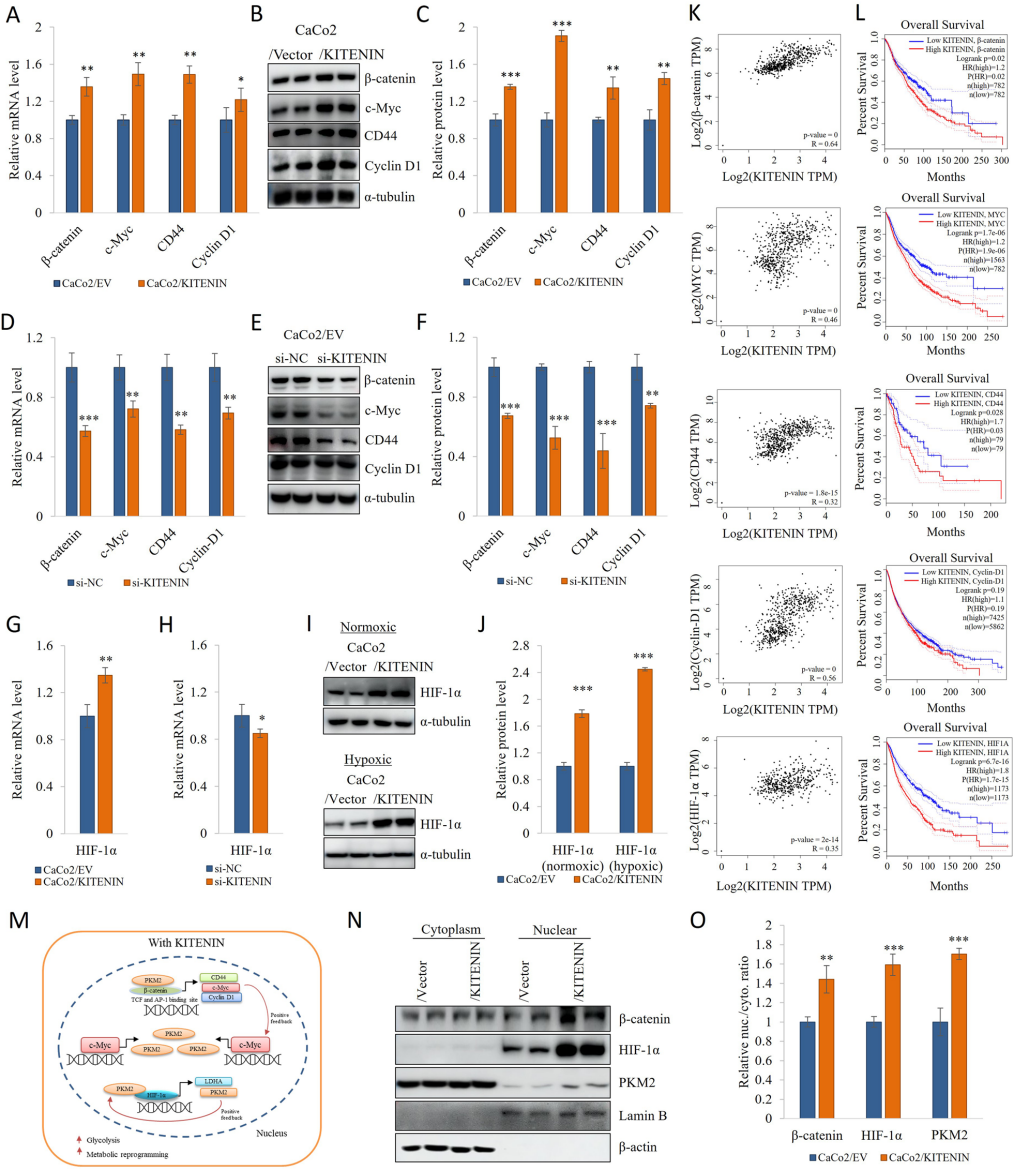
KITENIN promotes the downregulation of transcriptional regulators involved in aerobic glycolysis and of nuclear PKM2, β-catenin, and HIF-1α. To determine whether KITENIN overexpression or knockdown affects the expression of downstream effectors, their expression was analyzed using qRT-PCR and Western blotting. **A**–**C** mRNA expression and protein level of β-catenin, c-Myc, cyclin D1, and CD44 in CaCo2/EV and CaCo2/KITENIN cells. **D**–**F** KITENIN knockdown reduces the mRNA expression and protein level of β-catenin, c-Myc, cyclin D1, and CD44 in CaCo2/EV cells. **G** mRNA expression of HIF-1α in CaCo2/EV and CaCo2/KITENIN cells and **H** HIF-1α mRNA expression in KITENIN-silenced cells. **I**–**J** KITENIN increases HIF-1α protein level under both normoxic and hypoxic conditions in CaCo2/EV and CaCo2/KITENIN cells. **K** The GEPIA web tool was used to analyze the relationship of the mRNA expression of KITENIN with that of β-catenin, c-Myc, CD44, cyclin D1, and HIF-1α in COAD samples. **L** Overall survival curve of between KITENIN-β-catenin, KITENIN-MYC, KITENIN-CD44, KITENIN-CyclinD1, and KITENIN-HIF-1α levels, according to the GEPIA database. **M** Schematic illustration of how KITENIN increases nuclear PKM2, and PKM2 binds to β-catenin and HIF-1α, creating a feedback loop between c-Myc, PKM2, and HIF-1α. **N** KITENIN increases the nuclear localization of β-catenin, HIF-1α, and PKM2 in CaCo2/EV cells. Lamin B was used as a nuclear marker. **O** Nuclear-to-cytoplasmic ratios of β-catenin, HIF-1α, and PKM2 in CaCo2/EV and CaCo2/KITENIN cells. Data are the mean ± standard deviation, n = 3 * *p* < 0.05; ** *p* < 0.01; *** *p* < 0.001, NS, no significant difference between groups

PKM2 can translocate to the nucleus and promote transcriptional activity [51]. Furthermore, high expression of PKM2 was a feature of KITENIN-overexpressing cells. PKM2 forms a complex with ß-catenin, after which it can bind to the c-Myc and cyclin D1 promoters [52]. The TCF4 and AP-1 downstream gene CD44, a protein that is expressed on the cell surface in cancer stem cells, interact with PKM2, and thereby strengthen the glycolytic phenotype of hypoxic cancer cells [53, 54]. We found that the protein and mRNA expression of ß-catenin, c-Myc and cyclin D1 were increased by KITENIN-overexpressing conditions and reduced by KITENIN knockdown (Fig. 5A–F, Additional file 1: Fig S3E–F, S5G–H).

Although PKM2 functions as an enzyme in the glycolytic pathway, it also has non-enzymatic effects. The nuclear translocation of PKM2 permits it to act as a transcriptional activator of the HIF-1α and β-catenin genes [55–57], and PKM2/HIF-α promotes the expression of GLUT1 and LDHA. In addition, PKM2/β-catenin increases the expression of c-Myc and cyclin D1 [31, 33, 51]. In a previous study, it was shown that HIF-1α protein expression is lower in KITENIN knockdown cells and that the low HIF-1α expression affects angiogenesis [58]. In the present study, HIF-1α protein level was found to be high under both hypoxic and normoxic conditions in KITENIN-overexpressing. In addition, HIF-1α mRNA expression was high in KITENIN-overexpressing cells and lower in KITENIN-silenced cells (Fig. 5G–J, Additional file 1: Fig S3G, H, Fig S11A, B). Furthermore, using the GEPIA web tool, we found that the mRNA expression of KITENIN positively correlated with that of ß-catenin, c-Myc, cyclin D1, CD44, and HIF-α levels in colon cancer, in which their overexpression is associated with poor survival (Fig. 5K–L). We next measured the nuclear and cytoplasmic protein expression of β-catenin, HIF-1α, and PKM2 (Fig. 5M–O), which indicated greater translocation of PKM2 under KITENIN-overexpressing conditions (high nuclear/cytoplasmic ratios of β-catenin and HIF-1α). To further characterization, we showed that the knockdown of KITENIN and MYO1D downregulate β-catenin, cyclinD1 and HIF-1α levels in PKM2 overexpression cells (Additional file 1: Fig S11C–F).

### Disintegrator of KITENIN complex compounds reduce the KITENIN-induced upregulation of glycolysis

**Fig. 6:**
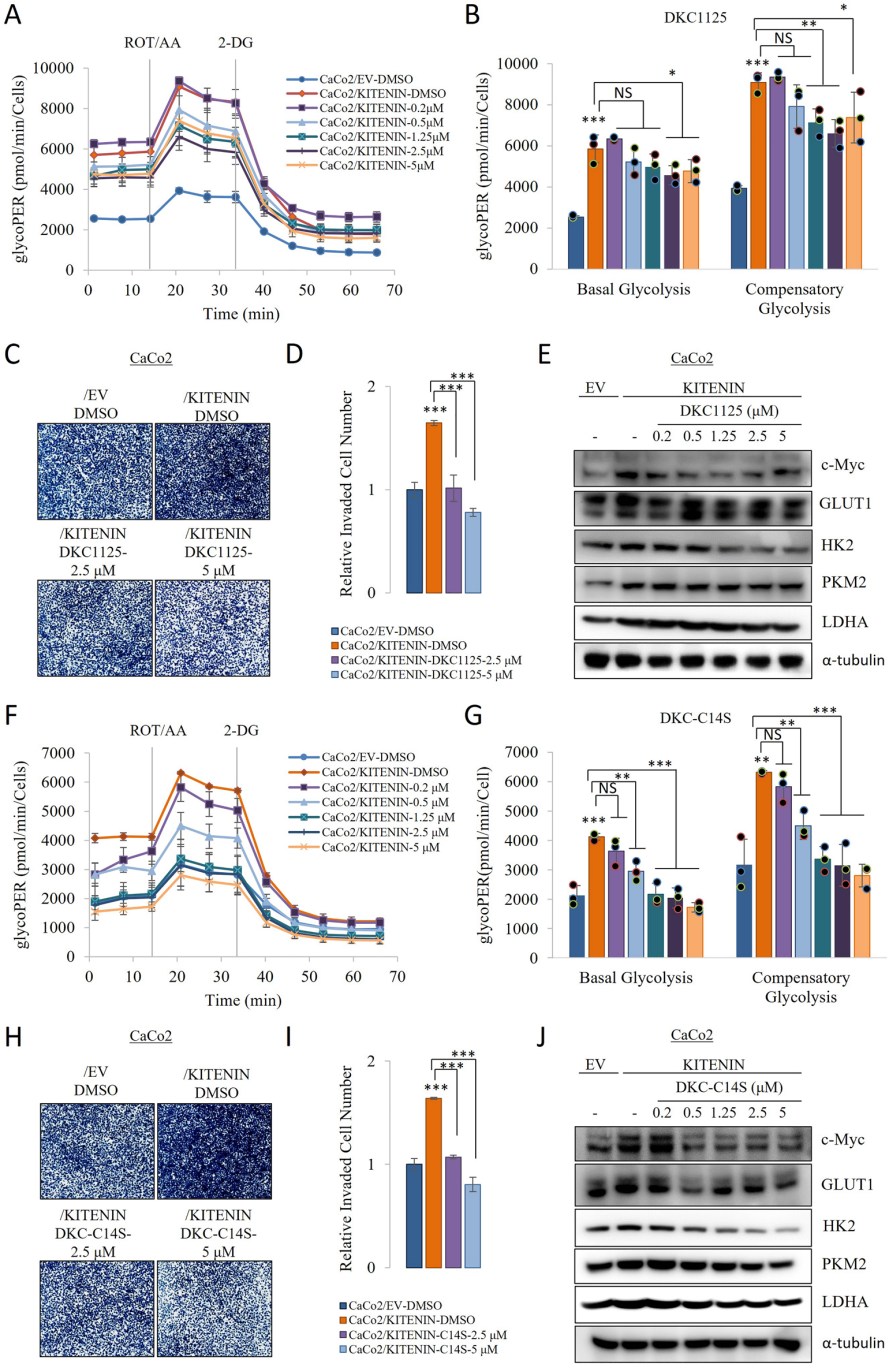
DKC1125 reduces the KITENIN-induced increase in aerobic glycolysis. **A** CaCo2/KITENIN cells were treated with the indicated concentrations of DKC1125 (0.2–5 µM) for 48 h. CaCo2/EV cells were used as controls. The extracellular acidification rate (ECAR) and oxygen consumption rate (OCR) were measured using a Glycolytic Rate Assay Kit on a Seahorse XF96 extracellular flux analyzer. To assess the glycolytic rate, the assay utilizes both ECAR and OCR measurements to determine the glycolytic proton efflux rate (glycoPER). GlycoPER was measured at two time points, and then following the sequential injection of rotenone (1 μM)/antimycin A (1 μM) and 2-DG (50 mM). **B** Basal glycolysis and compensatory glycolysis determined using the glycoPER curves. **C**–**D** Invasion assay for CaCo/EV and CaCo2/KITENIN cells, using fibronectin as a chemoattractant. KITENIN-overexpressing cells showed greater invasiveness than empty vector-transfected cells. Cells were treated with 2.5 and 5 μM of DKC1125 for 24 h. The stained invading cells were counted and the numbers in each group are shown in a bar graph. **E** Mechanism whereby DKC1125 reduces the KITENIN-induced increase in glycolysis. Protein levels of c-Myc, GLUT1, HK2, PKM2, and LDHA in CaCo2/EV and CaCo2/KITENIN cells incubated for 48 h. **F**-**G** CaCo2/KITENIN cells were treated with the indicated concentrations of DKC-C14S (0.2–5 µM) for 48 h. CaCo2/EV cells were used as controls. Basal glycolysis and compensatory glycolysis determined using the glycoPER curves. **H**–**I** Cells were treated with 2.5 and 5 μM of DKC-C14S for 24 h. The stained invading cells were counted and the numbers in each group are shown in a bar graph. **J** Mechanism whereby DKC-C14S reduces the KITENIN-induced increase in glycolysis. Protein expression of c-Myc, GLUT1, HK2, PKM2, and LDHA in CaCo2/EV and CaCo2/KITENIN cells incubated for 48 h. Data are the mean ± standard deviation, n = 3. * *p* < 0.05; ** *p* < 0.01; *** *p* < 0.001, NS, no significant difference between groups

In our previous study has been shown that DKC1125 prevents the formation of the functional KITENIN-KH-type splicing regulatory protein (KSRP)-RACK1-Dvl2 complex by binding to KSRP [18]. To determine the effects of DKC1125 on glycolysis in KITENIN-overexpressing cells, we measured the ECAR, and found that DKC1125 has a dose-dependent effect (Fig. 6A, B). Next, to identify the mechanism involved, we assessed the effects of DKC1125 on the protein expression of c-Myc, GLUT1, HK2, PKM2, and LDHA, and found dose-dependent reductions in the expression of each (Fig. 6E). Then we created the optimized DKC-C14S compound and compared it with the experiments we evaluated for DKC1125. Our results showed that DKC-C14S similarly suppressed cell invasion (Fig. 6C-D, H-I), while it was more effective on aerobic glycolysis (Fig. 6F, G, J). These results suggest that DKC compounds regresses aerobic glycolysis in cells with a functional KITENIN axis.

**Fig. 7:**
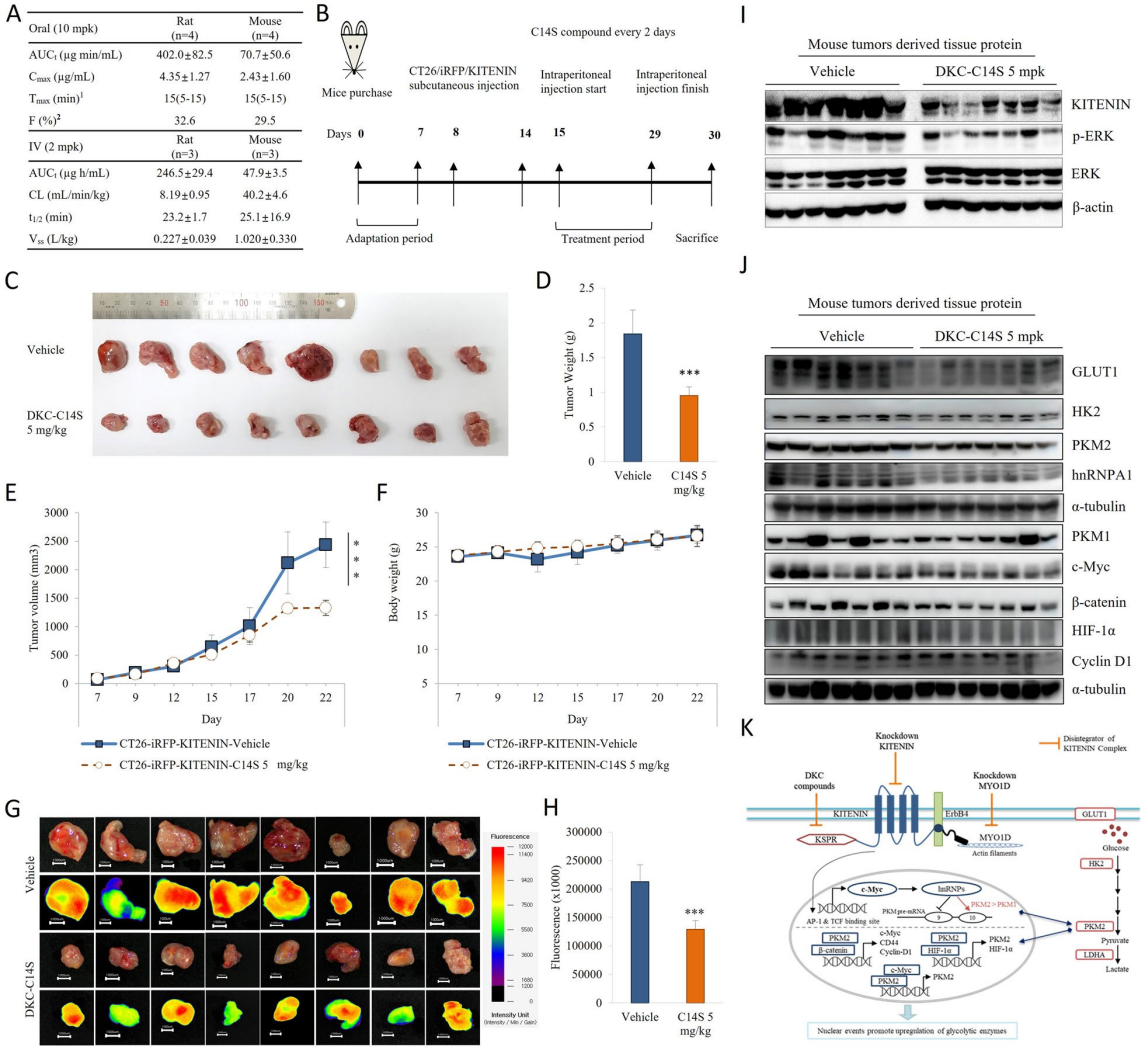
Treatment with soluble form of disintegrator of KITENIN complex, DKC-C14S the suppresses the tumor growth. **A** Pharmacokinetic parameters of DKC-C14S. AUC_t_: area under the curve from zero time to last sampling time; C_max_: maximum concentration; T_max_: time to reach C_max_; F: bioavailability; CL: total body clearance; t_1/2_: half-life; V_ss_: volume of distribution at steady state (^1^median (range) and ^2^F value was calculated as following equation; F(%) = 100xAUC_t_oral_/AUC_t_iv_). All data expressed as mean ± standard deviation. **B** Treatment of DKC-C14S significantly reduces the tumor formation in a tumor model with higher expression levels of KITENIN. 1 × 10^6^ CT-26/KITENIN-V5 cells were inoculated subcutaneously into BALB/c mice. After the tumors grew for 1 week, the DKC-C14S was given intravenously every other day for 14 days. **C**–**F** Mice were sacrificed on day 30 and images of tumors, tumors sizes, tumor weights and body weight in different treatment groups were acquired (mean ± SD). **G**–**H** Fluorescence area of images obtained from a fluorescence-labeled organism bioimaging instrument (FOBI) system (mean ± SD, n = 8). An asterisk indicates a significant difference between indicated groups. * *p* < 0.05; ** *p* < 0.01; *** *p* < 0.001, NS, no significant difference between groups. The decreased levels of KITENIN, ERK, metabolic marker and transcriptional regulators in tumor tissues given DKC-C14S. **I** KITENIN and its downstream signals protein ERK (total ERK and p-ERK) protein levels were investigated from the tumors tissues. **J** The protein levels of GLUT1, HK2, PKM1, PKM2, c-Myc, hnRNPA1, HIF-1α, β-catenin, and Cyclin D1 were examined via immunoblot analyses tumors collected from both groups, vehicle and DKC-C14S (5 mpk). **K** Schematic showing how the oncoprotein KITENIN affects metabolic and non-metabolic pathways regulating aerobic glycolysis. Overexpression of KITENIN upregulates the expression of the AP-1 and TCF4 target genes. Here, overexpression of c-Myc upregulates hnRNPI, hnRNPA1 and hnRNPA2 expressions, which are responsible for the PKM2/PKM1 ratio, and increases the PKM2/PKM1 ratio. All nuclear activities that occur with KITENIN overexpression upregulate the level of proteins responsible for aerobic glycolysis. Overexpressed in the cytoplasm PKM2, undergoes nuclear translocation, where it forms a complex with β-catenin and HIF-1a, feeding a positive feedback mechanism. The disintegration of the KITENIN complex by DKC compounds and the knockdowns inhibit KITENIN-mediated nuclear activity and upregulation of aerobic glycolysis

Later in this study, we created a skin xenograft model of DKC-C14S, the soluble form (Fig. 7A) of DKC1125, by inoculating CT26/KITENIN/iRFP cells into BALB/c mice (Fig. 7B). Tumor-bearing mice were administered control or DKC-C14S (5 mpk) intraperitoneally (i.p.) twice weekly beginning 1 week after inoculation. In comparison to the control group, the DKC-C14S therapy reduced tumor volume and weight by around 50–55%, as seen in Fig. 7C–H. To further confirmation, we examined the protein levels of KITENIN, ERK, p-ERK, metabolic marker and transcriptional regulators in the tumors collected from the both groups. DKC-C14S treatment disrupts the KITENIN and significantly reduces the protein level compared to the control group. As shown in Fig. 7I, KITENIN downstream signals phospho-ERK was reduced in the treatment group. Results showed the GLUT1, HK2, PKM2, PKM2/PKM1, HIF-1α, β-catenin, c-Myc, and hnRNPA1 levels downregulated by the DKC-C14S treatment, but CyclinD1 levels doesn’t change by the treatment (Fig. 7J, K). In Additional file 1: Fig S12, the quantitative amount of each protein was measured by densitometry and is represented as an arbitrary score (mean ± SD, n = 7).

## Discussion

KITENIN is an oncoprotein that is associated with the metastasis of tumors and a poor prognosis in patients. Specifically, it promotes invasiveness, early hepatic metastasis, angiogenesis, and tumorigenicity [8, 9, 13, 14, 58–60]. A number of mechanisms for these effects have been reported. KITENIN increases the migration of CRC cells in an ERK/AP-1 dependent manner [9]: KITENIN, Disheveled, and PKCδ form a complex that stimulates ERK/AP-1 and modulates invasiveness [9, 46]. The KITENIN/ErbB4-Dvl2-c-Jun axis is up-regulated by the EGF signal, independent of EGFR [12, 61]. KITENIN also promotes the expression of N-cadherin, ZEB1, ZEB2, SNAIL, and SLUG, which are markers of EMT; and CD133, CD44, ALDH1, and EPHB1, which are markers of “stemness” [11]; and cyclin D1, MMP-1, COX-2 genes [46]. In the present study, we assessed, for the first time, the relationship between the oncoprotein KITENIN and cancer metabolism.

Cellular metabolism has a key role in cancer and is an important area for research aimed at better understanding the molecular biology of this disease [62]. The identification of cancer-associated mutations in genes that encode metabolic enzymes demonstrated links between cancer and metabolism [63]. Indeed, many links between oncogenic signaling pathways and aspects of metabolism have been reported, and novel strategies have been used to identify relationships between oncogenes and metabolic vulnerabilities [64]. The metabolic reprogramming of cells is important for metastasis, apoptosis, the evasion of signals that inhibit cell growth, and uncontrolled cell proliferation [64, 65].

First, we analyzed the glycolysis and mitochondrial respiration of KITENIN-overexpressing cells and cells transfected with empty vector using a Seahorse XFe96 instrument and found that KITENIN-overexpressing cells predominantly use glycolysis for energy production. The level of mitochondrial respiration did not differ between KITENIN-overexpressing and empty vector-transfected cells. Next, we analyzed the mechanism of the effect of KITENIN on aerobic glycolysis. Glucose is catabolized to pyruvate and lactic acid during glycolysis, with very little ATP being produced [66]. The activity of the glycolytic pathway is largely regulated through GLUT1 and the enzymes HK2, PKM2, and LDHA [67]. We found that there was higher mRNA expression and protein level of all of these molecules in KITENIN-overexpressing cells (Fig. 1).

The transport of glucose across cell membranes is mediated by facilitative and energy-dependent mechanisms involving the GLUT family or sodium/glucose-linked transporter family [68]. GLUT1 is responsible for the transport of monosaccharides such as glucose, galactose, mannose, and glucosamine [69], and HKs are responsible for the intracellular phosphorylation of glucose to form glucose-6-phosphate. [70]. PKM2 uses ADP and phosphoenolpyruvate (PEP) to generate ATP and pyruvate [71, 72], and this enzyme is activated by fructose-1,6-bisphosphate (FBP); therefore, an increase in FBP concentration causes an increase in PKM2 activity and increases the rate of glycolysis [73–76]. Two isomers of LDH, LDHA and LDHB, are responsible for the final step of the glycolytic pathway: the conversion of pyruvate to lactate [77]. High expression of both PKM2 and LDHA has previously been shown in cancer cells, and promotes tumor progression [78, 79].

RTKs frequently regulate differentiation, cell growth, and survival, and are regarded as proto-oncoproteins because the overexpression or activation of many are associated with the development and growth of tumors [42–44]. In our previous study, we found that MYO1D knockdown causes RTKs such as those in the EGFR family, including KITENIN and ErbB4, to detach from the plasma membrane, such that they can be proteasomal degraded in the cytosol [27]. In the present study, we have shown that MYO1D knockdown downregulates KITENIN and ErbB4 mRNA expression and that glycoPER and cell cycle activity on KITENIN overexpression. Cell division is an energy-requiring process and metabolism plays an important role in the cell cycle [52]. To further characterize, we investigated the effect of KITENIN knockdown on glycolysis and glycolytic enzyme expression and found a suppression of aerobic glycolysis (Figs. 2, 3, and Additional file 1: Fig S5).

The oncoprotein c-Myc promotes the transcription of hnRNPI, hnRNPA1, and hnRNPA2, which reduce PKM1 mRNA expression by binding to the RNA sequence encoded by exon 9, resulting in greater PKM2 (exon 10) isoform expression [29, 30]. Here, we found that the PKM2/PKM1 ratio was affected by the expression of KITENIN (Figs. 1, 4, 5). PKM2 is an enzyme of the glycolytic pathway, but also an active protein kinase that plays a role in oncogenic signal transduction and overexpression of PKM2 upgrades the progression of malignancy in colorectal cancer [80]. In addition, new therapeutic approaches to inhibiting the NOX4/PKM2-dependent immunometabolism pathway have been reported as potential targets in the treatment of sepsis and inflammatory diseases [81]. Another report reported that extracellular matrix PKM2 facilitates the progression of organ fibrosis, promotes apoptosis resistance and collagen production in myofibroblasts, and activates the αvβ3-FAK-PI3K signal axis. It has also been shown to facilitate angiogenesis of extracellular maxtrix PKM2 in cancer cells [82]. The findings from another study, which identified important prospective transcriptome indicators and biological pathways in hyperglycemic HK-2 cells responsive to the PKM2 activator TEPP-46, underscore the need for more investigation into the signaling mechanisms regulating EGFR and metabolic stress [83].

Transcriptional regulators affect the initiation and progression of cancer, including metastasis and cellular proliferation [84]. Nuclear PKM2 regulates β-catenin processing, which affects EGFR activation, and cyclin D1 and c-Myc expression is upregulated by the binding of β-catenin to the promoter region of the appropriate genes. In addition, ß-catenin, c-Myc, and cyclin D1 expression is increased by PKM2 transactivation [51]. Furthermore, PKM2 promotes the transcriptional activity of HIF-1α and PKM2 is a target gene of HIF-1α [30]. HIF-1α is activated in cancer, because of genetic defects, or in low oxygen tensions, and is responsible for the transition from oxidative phosphorylation to aerobic glycolysis [86–88]. KITENIN overexpression increases cell migration via mitogen-activated protein kinase (MAPK)/ERK/AP-1 signaling [88, 89]. The induction of HIF-1α via the Raf/MEK/ERK pathway has also been shown previously and there are studies evaluating the compound activities for inhibition of Raf/MEK/ERK signaling pathway induced by HIF-1α [90, 91].

Changes in aerobic glycolysis may be important for metastasis and other effects of the KITENIN oncoprotein. Previous studies have shown that miR-124 targets KITENIN and suppresses tumorigenesis [13], and suppressed the PKM2 by inhibition of hnRNPs [41]. The results of these two previous studies that assessed the role of miR-124 are consistent with the present findings. To summarize these mechanical changes briefly, it shows that KITENIN regulates the metabolic pathway by defining a new oncogenic function. We markedly identify PKM2 signaling pathway activation downstream of KITENIN. Metabolic changes and their regulation are very complex and multiple processes [92]. Reports that PKM2 expression can be induced by binding to the PKM promoter region of c-Myc [93]. Another study showed that c-Myc pre-mRNA and mature mRNA were not altered in PKM2-destroyed cells in reverse transcription polymerase chain reaction (RT-PCR) analysis [76]. There have been reports that c-Myc and PKM2 expressions work within a feedback mechanism and can regulate each other [94]. In the current study, we have shown that KITENIN overexpressed cells selectively promote M2 from PKM isoforms, supporting the literature research suggesting that the c-Myc/hnRNP axis is involved in the initiation of this process. We also present evidence for the interaction of c-Myc with PKM2 in KITENIN overexpressed cells. Here, we investigated how PKM2-mediated changes in aerobic glycolysis are regulated based on KITENIN expression and we suggest that it regulated by the c-Myc/hnRNPs axis. As a result of the different regulatory factors here, further investigation of the interactions between cellular processes is needed.

In our previous study, we developed a KSRP-binding compound that modifies KITENIN expression in multiple types of cancer, and this was developed as a strategy to reduce distant metastasis and chemoresistance. We showed that KITENIN-phospho-Dvl2 and the AP-1/c-jun axis signaling pathway are inhibited by DKC1125 treatment [18]. Therefore, finally, we determined whether DKC1125 and DKC-C14S would inhibit the effect of KITENIN on aerobic glycolysis and found that it reduced glycolysis.

## Conclusions

In the present study, we have demonstrated a regulatory role for KITENIN in aerobic glycolysis. c-Myc regulates the expression of hnRNP isoforms, and their upregulation increases the expression of PKM2, which transactivates the genes expressing β-catenin, c-Myc, cyclin D1, CD44, and HIF-1α in KITENIN-overexpressing cells. MYO1D supports KITENIN-induced aerobic glycolysis by regulating the expression and activation of the ErbB4/KITENIN complex by enabling the cell surface receptor ErbB4 anchor them to plasma membrane. These changes in signaling that occur secondary to KITENIN expression enable the upregulation of aerobic glycolysis, resulting in poor survival. Furthermore, DKC compounds inhibit the KITENIN-induced upregulation of aerobic glycolysis and tumorigenesis. Thus, we have shed light on the mechanism of metabolic reprogramming in cancer, and this may lead to the identification of novel target molecules for its treatment.

### Supplementary Information

**Additional file 1: Figure S1.** The overexpression of KITENIN (VANGL1) is correlated with unfavorable prognosis in malignant tumors. (A) The gene expression profile across all tumor samples and paired normal tissues. (B) VANGL1 and transcriptional regulators regulating the metabolic pathway are highly regulated in colon cancer. VANGL1 and transcriptional regulators mRNA expression level determined by the GEPIA web tool. The boxplot analysis showed the expression level by log2 (TPM + 1) on a log-scale. ns, not significant; *, P < 0.05. **Figure S2.** KITENIN(VANGL1) and genes involved in the aerobic glucose pathway are correlated. The GEPIA web tool was searched for the correlation between the expression of (A) VANGL1-SLC2A1 (GLUT1), (C) VANGL1-HK2, (E) VANGL1-LDHA and (G) VANGL1-SLC16A1 in mRNA levels in COAD samples. Survival rates between high and low expression of (B) VANGL1-GLUT1, (D) VANGL1-HK2, (F) VANGL1-LDHA and (H) VANGL1-MCT1 levels from GEPIA database. **Figure S3.** Overexpression KITENIN induce the aerobic glucose on CT26 murine CRC cell line. (A-B) CT26/EV and CT26/KITENIN cells incubated 48h and then examinated for metabolism assays. The extracellular acidification rate (ECAR) and oxygen consumption rate (OCR) were detected using the Glycolytic Rate Assay Kit on a Seahorse XF96 extracellular flux analyzer. To measure glycolytic rates, the assay utilizes both ECAR and OCR measurements to determine the glycolytic proton efflux rate (glycoPER). GlycoPER was measured at two time points, followed by sequential injection of rotenone (1 μM)/antimycin A (1 μM), and 2-DG (50 mM). (C-D) To elucidate the mechanisms by KITENIN on glycolysis pathways. Relative protein expression of Warburg effect responsible key enzymes (GLUT1, HK2, PKM1, PKM2) in CT26/EV and CT26/KITENIN cells incubated for 48h. (E-F) Downstream effectors analyzed by Western blotting on KITENIN overexpression condition in CT26/EV and CT26/KITENIN cells. KITENIN induces the β-catenin, c-myc, and CyclinD1 protein level. (G-H) Hypoxia inducible factor-1α induce on KITENIN overexpression condition. α-tubulin or actin served as a loading control. Data represent means ± standard deviation. * p < 0.05; ** p < 0.01; *** p < 0.001. **Figure S4.** Relative protein expression of Warburg effect responsible key enzymes (GLUT1, HK2, PKM2, LDHA) under hypoxic condition on CaCo2/EV and CaCo2/KITENIN cells. Data represent means ± standard deviation. * p < 0.05; ** p < 0.01; *** p < 0.001. **Figure S5.** KITENIN knockdown reduced the Warburg effect and responsible key enzymes on HCT116 cell line. (A-B) HCT116 cells transfected si-KITENIN and then examined for metabolism assays. The extracellular acidification rate (ECAR) and oxygen consumption rate (OCR) were detected using the Glycolytic Rate Assay Kit on a Seahorse XF96 extracellular flux analyzer. To measure glycolytic rates, the assay utilizes both ECAR and OCR measurements to determine the glycolytic proton efflux rate (glycoPER). glycoPER was measured at two time points, followed by sequential injection of rotenone (1 μM)/antimycin A (1 μM), and 2-DG (50 mM). (C) KITENIN knockdown efficiency control on mRNA level of KITENIN and ErbB4. (D) Relative mRNA of Warburg effect responsible key enzymes (GLUT1, HK2, PKM1, PKM2 LDHA) in HCT116 cells incubated for 48h. (E-F) Protein level of the GLUT1, HK2, PKM1, PKM2 on KITENIN knockdown condition. (G-H) β-catenin, CD44 and Cyclin D1 protein levels on KITENIN knockdown condition. Data represent means ± standard deviation. * p < 0.05; ** p < 0.01; *** p < 0.001. **Figure S6.** MYO1D knockdown reduced the KITENIN, Warburg effect key enzymes and c-Myc/hnRNPs protein expressions on CaCo2 cell line. CaCo2 cells transfected with si-MYO1D for 24h and then examined for Western blotting. (A-B) KITENIN, (C-D) GLUT1, HK2, PKM2 and LDHA, (E-F) c-Myc, hnRNPA1 and hnRNPA2 protein levels. Data represent means ± standard deviation. * p < 0.05; ** p < 0.01; *** p < 0.001. **Figure S7.** Flow cytometric analysis of the cell cycle distributions of Caco2/EV and CaCo2/KITENIN cells. (A–B) Cells were transfected with si-control or si-MYO1D and then incubated for 24 h, after which cells in the subG1, G1, S, and G2M phases were quantified. (C) Effects of si-MYO1D on the mRNA expression of MYO1D, assessed using two different primer sequences (MYO1D #1 and MYO1D #2), in CaCo2/EV and CaCo2/KITENIN cells. * p < 0.05; ** p < 0.01; *** p < 0.001. **Figure S8.** Effect of si-PKM2 transfection on the level of PKM2, PKM1, and KITENIN proteins in CaCo2/EV and CaCo2/KITENIN cells. (A, B) PKM2, PKM1, and KITENIN levels. β-Actin or GAPDH served as the loading control. **Figure S9.** (A) KITENIN overexpression enhances c-Myc-PKM2 interaction. CaCo2/EV and CaCo2/KITENIN cells were immunoprecipitated with anti-c-Myc antibody and immunoblotted with the indicated antibodies. (B) Calculation of PKM2/c-Myc IPed was given relative to Caco/EV. * p < 0.05. **Figure S10.** Knockdown of KITENIN and MYO1D inhibit c-Myc and hnRNPI, hnRNPA1, hnRNPA2 mRNA levels on PKM2-myc transfected cells. HCT116 cells were transfected with si-KITENIN and si-MYO1D for 24h followed by transfection of the plasmid, a construct expressing myc-tagged PKM2 (PKM2-myc) for 24h and subjected to the qRT-PCR. Data represent means ± standard deviation, n = 3. * p < 0.05; ** p < 0.01; *** p < 0.001. **Figure S11.** KITENIN overexpression is induced under hypoxic conditions treatment with 300 μM Cobalt (II) chloride (CoCl2) on CaCo2/EV and CaCo2/KITENIN cells. (A) Normoxic condition and hypoxic condition on the same blotting membrane, HIF-1A level. (B) Relative protein level of HIF-1α. (C-F) CaCo2 and HCT116 cells were transfected with si-KITENIN and si-MYO1D for 24h followed by transfection of the plasmid, a construct expressing myc-tagged PKM2 (PKM2-myc) for 24h and subjected to the β-catenin, CyclinD1 and HIF-1α mRNA levels. Data represent means ± standard deviation. * p < 0.05; ** p < 0.01; *** p < 0.001. **Figure S12.** KITENIN and its downstream signals protein ERK (total ERK and p-ERK), GLUT1, HK2, PKM1, PKM2, c-Myc, hnRNPA1, HIF-1α, β-catenin, and Cyclin D1 were examined via immunoblot analyses tumors collected from both groups, vehicle and DKC-C14S (5 mpk). The quantitative amount of each protein was measured by densitometry and is represented as an arbitrary score (mean±SD, n=7). An asterisk indicates a significant difference between indicated groups. * p < 0.05; ** p < 0.01; *** p < 0.001, NS, no significant difference between groups. **Table S1.** Primer (Forward/Reverse) sequences. **Table S2.** Antibodies information.

## Data Availability

All the data generated or analyzed during this study are included in this published article and its Additional files.

## Abbreviations

AP-1: Activator protein 1
CRC: Colorectal cancer
ECAR: Extracellular acidification rate
EGF: Epidermal growth factor
ERK: Extracellular signal-regulated kinase
HIF-1α: Hypoxia-inducible factor 1-alpha
glycoPER: Glycolytic proton flux rate
GLUT1: Glucose transporter 1
HK2: Hexokinase 2
HnRNP: Heterogeneous RNP
OCR: Oxygen comsumption rate
KITENIN: KAI1 COOH-terminal interacting tetraspanin
LDHA: Lactate dehydrogenase A
MAPK: Mitogen-activated protein kinase
MYO1D: Myosin ID
PEP: Phosphoenolpyruvate
PKC: Protein kinase C
PKM: Pyruvate kinase M
TCA: Tricarboxylic cycle
TPM: Transcripts per million
VANGL1: Van Gogh-like protein 1
