# Supplementary material for: KITENIN promotes aerobic glycolysis through PKM2 induction by upregulating the c-Myc/hnRNPs axis in colorectal cancer
Source: Cell Biosci. 2023 Aug 8;13:146. doi: 10.1186/s13578-023-01089-1 (PMC10410973; doi:10.1186/s13578-023-01089-1)
Supplement: Supplementary file 1 — Additional file 1: Figure S1. The overexpression of KITENIN (VANGL1) is correlated with unfavorable prognosis in malignant tumors. (A) The gene expression profile across all tumor samples and paired normal tissues. (B) VANGL1 and transcriptional regulators regulating the metabolic pathway are highly regulated in colon cancer. VANGL1 and transcriptional regulators mRNA expression level determined by the GEPIA web tool. The boxplot analysis showed the expression level by log2 (TPM + 1) on a log-scale. ns, not significant; *, P < 0.05. Figure S2. KITENIN(VANGL1) and genes involved in the aerobic glucose pathway are correlated. The GEPIA web tool was searched for the correlation between the expression of (A) VANGL1-SLC2A1 (GLUT1), (C) VANGL1-HK2, (E) VANGL1-LDHA and (G) VANGL1-SLC16A1 in mRNA levels in COAD samples. Survival rates between high and low expression of (B) VANGL1-GLUT1, (D) VANGL1-HK2, (F) VANGL1-LDHA and (H) VANGL1-MCT1 levels from GEPIA database. Figure S3. Overexpression KITENIN induce the aerobic glucose on CT26 murine CRC cell line. (A-B) CT26/EV and CT26/KITENIN cells incubated 48h and then examinated for metabolism assays. The extracellular acidification rate (ECAR) and oxygen consumption rate (OCR) were detected using the Glycolytic Rate Assay Kit on a Seahorse XF96 extracellular flux analyzer. To measure glycolytic rates, the assay utilizes both ECAR and OCR measurements to determine the glycolytic proton efflux rate (glycoPER). GlycoPER was measured at two time points, followed by sequential injection of rotenone (1 μM)/antimycin A (1 μM), and 2-DG (50 mM). (C-D) To elucidate the mechanisms by KITENIN on glycolysis pathways. Relative protein expression of Warburg effect responsible key enzymes (GLUT1, HK2, PKM1, PKM2) in CT26/EV and CT26/KITENIN cells incubated for 48h. (E-F) Downstream effectors analyzed by Western blotting on KITENIN overexpression condition in CT26/EV and CT26/KITENIN cells. KITENIN induces the β-catenin, c-myc, and CyclinD1 pr [file 13578_2023_1089_MOESM1_ESM.docx]

**Additional file Information**

**KITENIN promotes aerobic glycolysis through PKM2 induction by upregulating the c-Myc/hnRNPs axis in colorectal cancer**

Running Title: KITENIN promotes aerobic glycolysis

Mücahit Varlı ^1^, Sung Jin Kim ^1,2^, Myung-Giun Noh ^3^, Yoon Gyoon Kim^4^, Hyung-Ho Ha ^1^, Kyung Keun Kim ^2^, Hangun Kim ^1,^*

^1^College of Pharmacy, Sunchon National University, 255 Jungang-ro, Sunchon 57922, Republic of Korea

^2^Department of Pharmacology, Chonnam National University Medical School, 160 Baekseoro, Dong-gu, Gwangju, 61469, Republic of Korea

^3^Department of Pathology, Chonnam National University Medical School, 160 Baekseoro, Dong-gu, Gwangju, 61469, Republic of Korea

^4^ College of Pharmacy, Dankook University, 119 Dandaero, Dongnam-gu, Cheonan-si 31116, Republic of Korea

Correspondence:

^*^College of Pharmacy, Sunchon National University,

255 Jungang-ro, Sunchon, Jeonnam 57922, Korea

hangunkim@sunchon.ac.kr; Tel.: +82-61-750-3761

**Additional file 1: Supplementary Figure S1.** The overexpression of KITENIN (VANGL1) is correlated with unfavorable prognosis in malignant tumors. (A) The gene expression profile across all tumor samples and paired normal tissues. (B) VANGL1 and transcriptional regulators regulating the metabolic pathway are highly regulated in colon cancer. VANGL1 and transcriptional regulators mRNA expression level determined by the GEPIA web tool. The boxplot analysis showed the expression level by log2 (TPM + 1) on a log-scale. ns, not significant; *, P < 0.05.

**Additional file 1:** **Supplementary Figure S2.** KITENIN(VANGL1) and genes involved in the aerobic glucose pathway are correlated. The GEPIA web tool was searched for the correlation between the expression of (A) VANGL1-SLC2A1 (GLUT1), (C) VANGL1-HK2, (E) VANGL1-LDHA and (G) VANGL1-SLC16A1 in mRNA levels in COAD samples. Survival rates between high and low expression of (B) VANGL1-GLUT1, (D) VANGL1-HK2, (F) VANGL1-LDHA and (H) VANGL1-MCT1 levels from GEPIA database.

**Additional file 1: Supplementary Figure S3.** Overexpression KITENIN induce the aerobic glucose on CT26 murine CRC cell line. (A-B) CT26/EV and CT26/KITENIN cells incubated 48h and then examinated for metabolism assays. The extracellular acidification rate (ECAR) and oxygen consumption rate (OCR) were detected using the Glycolytic Rate Assay Kit on a Seahorse XF96 extracellular flux analyzer. To measure glycolytic rates, the assay utilizes both ECAR and OCR measurements to determine the glycolytic proton efflux rate (glycoPER). GlycoPER was measured at two time points, followed by sequential injection of rotenone (1 μM)/antimycin A (1 μM), and 2-DG (50 mM). (C-D) To elucidate the mechanisms by KITENIN on glycolysis pathways. Relative protein expression of Warburg effect responsible key enzymes (GLUT1, HK2, PKM1, PKM2) in CT26/EV and CT26/KITENIN cells incubated for 48h. (E-F) Downstream effectors analyzed by Western blotting on KITENIN overexpression condition in CT26/EV and CT26/KITENIN cells. KITENIN induces the β-catenin, c-myc, and CyclinD1 protein level. (G-H) Hypoxia inducible factor-1α induce on KITENIN overexpression condition. ɑ-tubulin or actin served as a loading control. Data represent means ± standard deviation. * p < 0.05; ** p < 0.01; *** p < 0.001.

**Additional file 1: Supplementary Figure S4.** Relative protein expression of Warburg effect responsible key enzymes (GLUT1, HK2, PKM2, LDHA) under hypoxic condition on CaCo2/EV and CaCo2/KITENIN cells. Data represent means ± standard deviation. * p < 0.05; ** p < 0.01; *** p < 0.001.

**Additional file 1: Supplementary Figure S5.** KITENIN knockdown reduced the Warburg effect and responsible key enzymes on HCT116 cell line. (A-B) HCT116 cells transfected si-KITENIN and then examined for metabolism assays. The extracellular acidification rate (ECAR) and oxygen consumption rate (OCR) were detected using the Glycolytic Rate Assay Kit on a Seahorse XF96 extracellular flux analyzer. To measure glycolytic rates, the assay utilizes both ECAR and OCR measurements to determine the glycolytic proton efflux rate (glycoPER). glycoPER was measured at two time points, followed by sequential injection of rotenone (1 μM)/antimycin A (1 μM), and 2-DG (50 mM). (C) KITENIN knockdown efficiency control on mRNA level of KITENIN and ErbB4. (D) Relative mRNA of Warburg effect responsible key enzymes (GLUT1, HK2, PKM1, PKM2, LDHA) in HCT116 cells incubated for 48h. (E-F) Protein level of the GLUT1, HK2, PKM1, PKM2 on KITENIN knockdown condition. (G-H) β-catenin, CD44 and Cyclin D1 protein levels on KITENIN knockdown condition. Data represent means ± standard deviation. * p < 0.05; ** p < 0.01; *** p < 0.001.

**Additional file 1: Supplementary Figure S6.** MYO1D knockdown reduced the KITENIN, Warburg effect key enzymes and c-Myc/hnRNPs protein expressions on CaCo2 cell line. CaCo2 cells transfected with si-MYO1D for 24h and then examined for Western blotting. (A-B) KITENIN, (C-D) GLUT1, HK2, PKM2 and LDHA, (E-F) c-Myc, hnRNPA1 and hnRNPA2 protein levels. Data represent means ± standard deviation. * p < 0.05; ** p < 0.01; *** p < 0.001.

**Additional file 1: Supplementary Figure S7.** **Flow cytometric analysis of the cell cycle distributions of Caco2/EV and CaCo2/KITENIN cells.** (A–B) Cells were transfected with si-control or si-MYO1D and then incubated for 24 h, after which cells in the subG1, G1, S, and G2M phases were quantified. (C) Effects of si-MYO1D on the mRNA expression of MYO1D, assessed using two different primer sequences (MYO1D #1 and MYO1D #2), in CaCo2/EV and CaCo2/KITENIN cells. * p < 0.05; ** p < 0.01; *** p < 0.001.

**Additional file 1: Supplementary Figure S8.** Effect of si-PKM2 transfection on the level of PKM2, PKM1, and KITENIN proteins in CaCo2/EV and CaCo2/KITENIN cells. (A, B) PKM2, PKM1, and KITENIN levels. β-Actin or GAPDH served as the loading control.

**Additional file 1:** **Supplementary Figure S9.** (A) KITENIN overexpression enhances c-Myc-PKM2 interaction. CaCo2/EV and CaCo2/KITENIN cells were immunoprecipitated with anti-c-Myc antibody and immunoblotted with the indicated antibodies. (B) Calculation of PKM2/c-Myc IPed was given relative to Caco/EV. * p < 0.05

**Additional file 1: Supplementary Figure S10.** Knockdown of KITENIN and MYO1D inhibit c-Myc and hnRNPI, hnRNPA1, hnRNPA2 mRNA expression on PKM2-myc transfected cells. HCT116 cells were transfected with si-KITENIN and si-MYO1D for 24h followed by transfection of the plasmid, a construct expressing myc-tagged PKM2 (PKM2-myc) for 24h and subjected to the qRT-PCR. Data represent means ± standard deviation, n = 3. * p < 0.05; ** p < 0.01; *** p < 0.001.

**Additional file 1: Supplementary Figure S11.** KITENIN overexpression is induced under hypoxic conditions treatment with 300 μM Cobalt (II) chloride (CoCl2) on CaCo2/EV and CaCo2/KITENIN cells. (A) Normoxic condition and hypoxic condition on the same blotting membrane, HIF-1A level. (B) Relative protein level of HIF-1α. (C-F) CaCo2 and HCT116 cells were transfected with si-KITENIN and si-MYO1D for 24h followed by transfection of the plasmid, a construct expressing myc-tagged PKM2 (PKM2-myc) for 24h and subjected to the β-catenin, CyclinD1 and HIF-1α mRNA levels. Data represent means ± standard deviation. * p < 0.05; ** p < 0.01; *** p < 0.001.

**Additional file 1: Supplementary Figure S12.** KITENIN and its downstream signals protein ERK (total ERK and p-ERK), GLUT1, HK2, PKM1, PKM2, c-Myc, hnRNPA1, HIF-1α, β-catenin, and Cyclin D1 were examined via immunoblot analyses tumors collected from both groups, vehicle and DKC-C14S (5 mpk). The quantitative amount of each protein was measured by densitometry and is represented as an arbitrary score (mean±SD, n=7). An asterisk indicates a significant difference between indicated groups. * *p*< 0.05; ** *p*< 0.01; *** *p*< 0.001, NS, no significant difference between groups.

**Additional file 1: Supplementary Table S1.** Primer (Forward/Reverse) sequences.

**Additional file 1: Supplementary Table S2.** Antibodies information.

**
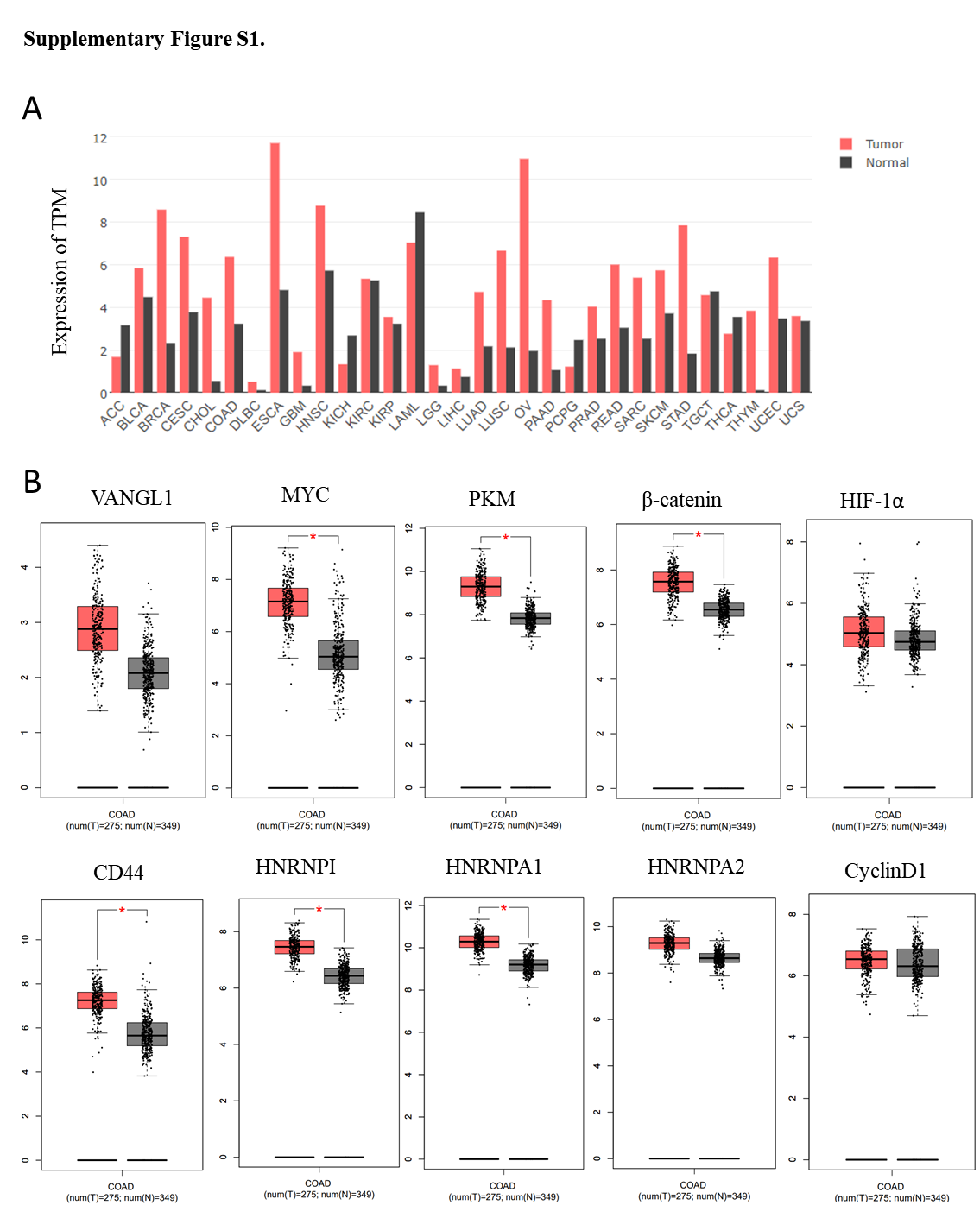
**

**
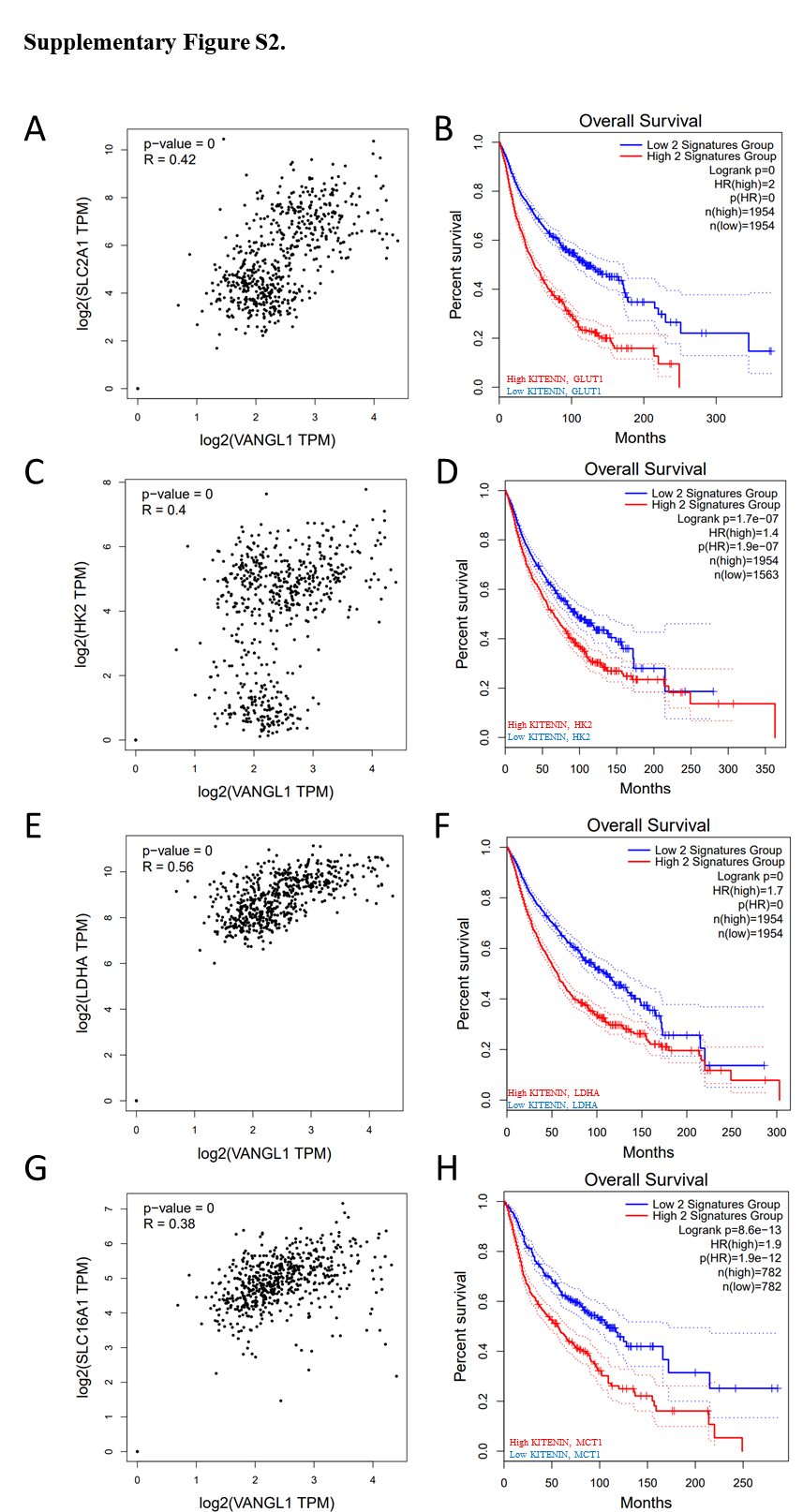
**

**
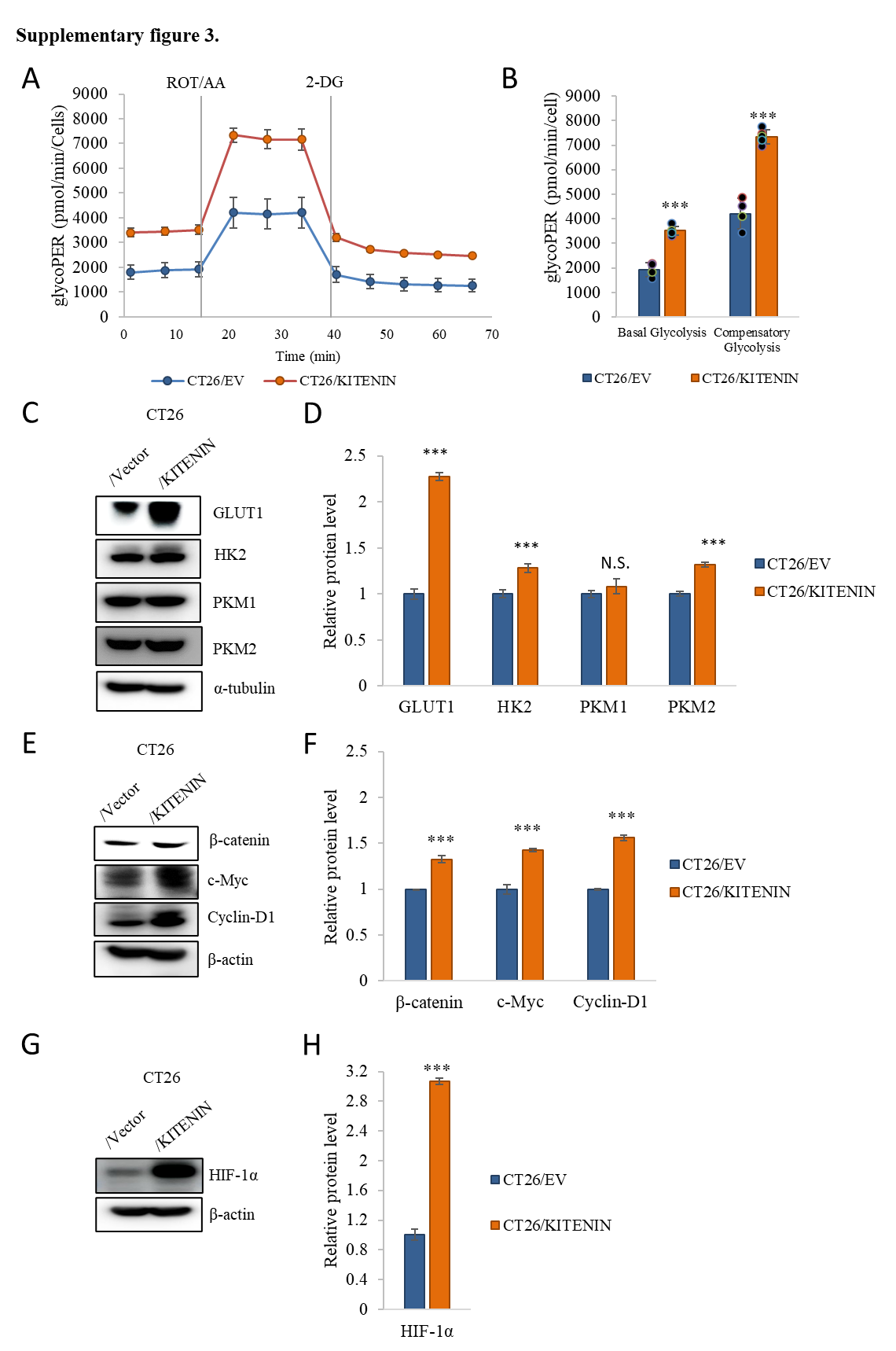
**

**
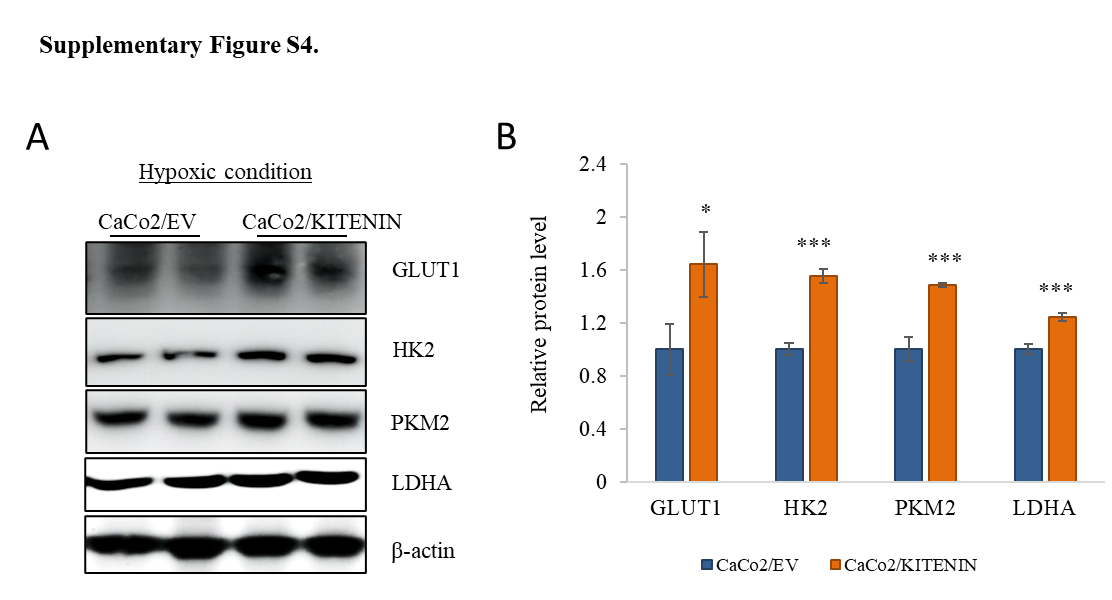
**

**
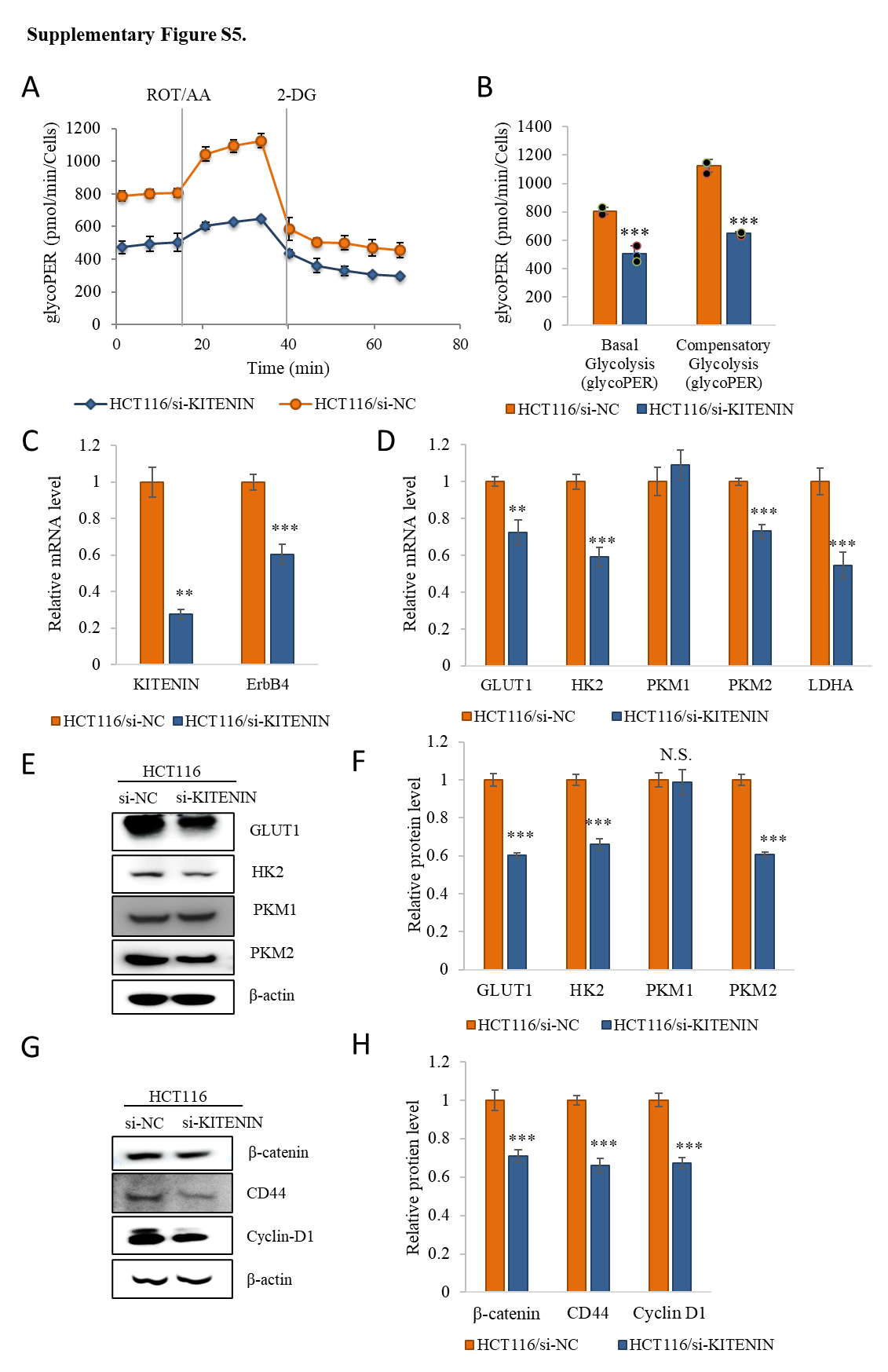
**

**
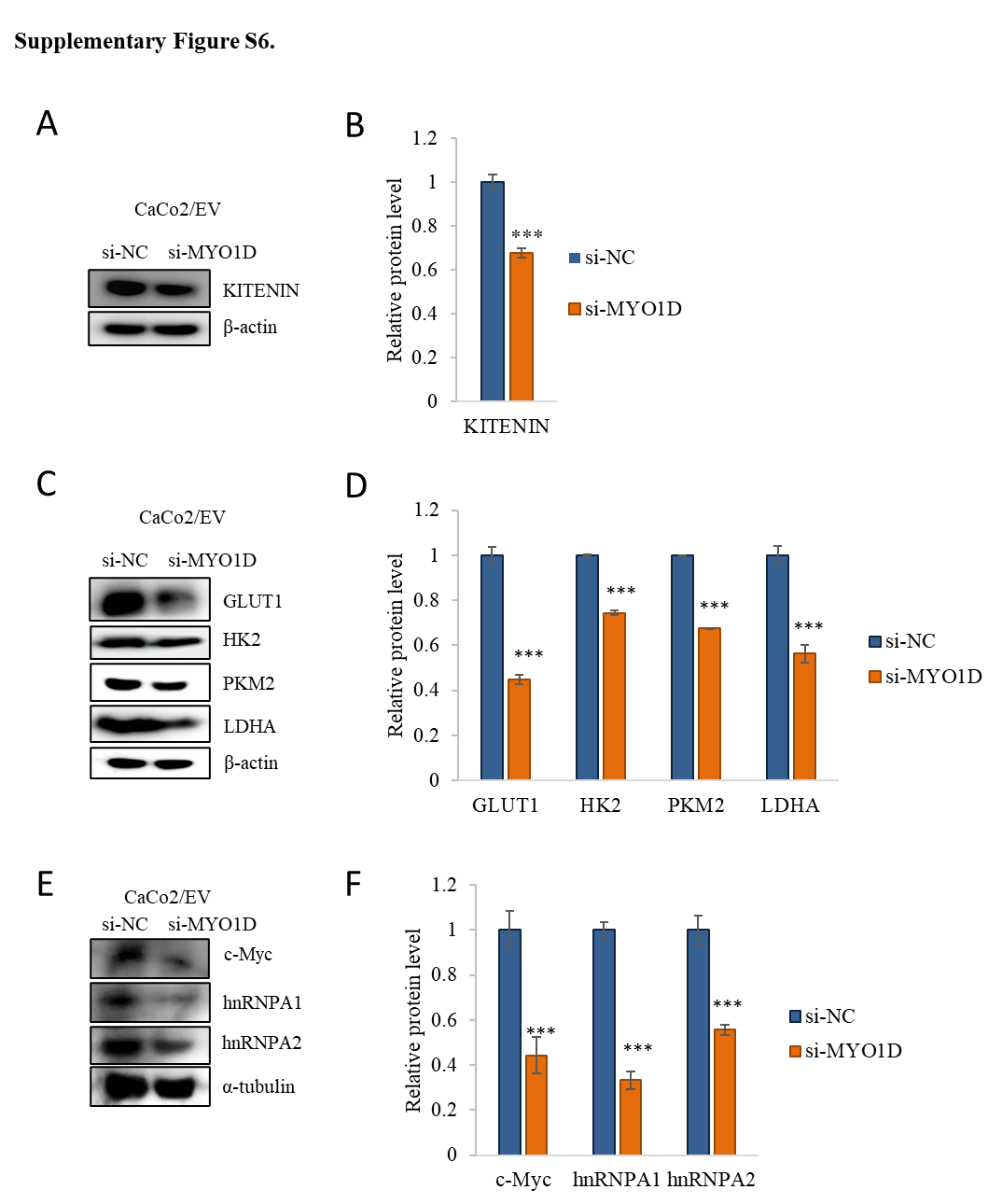
**

**
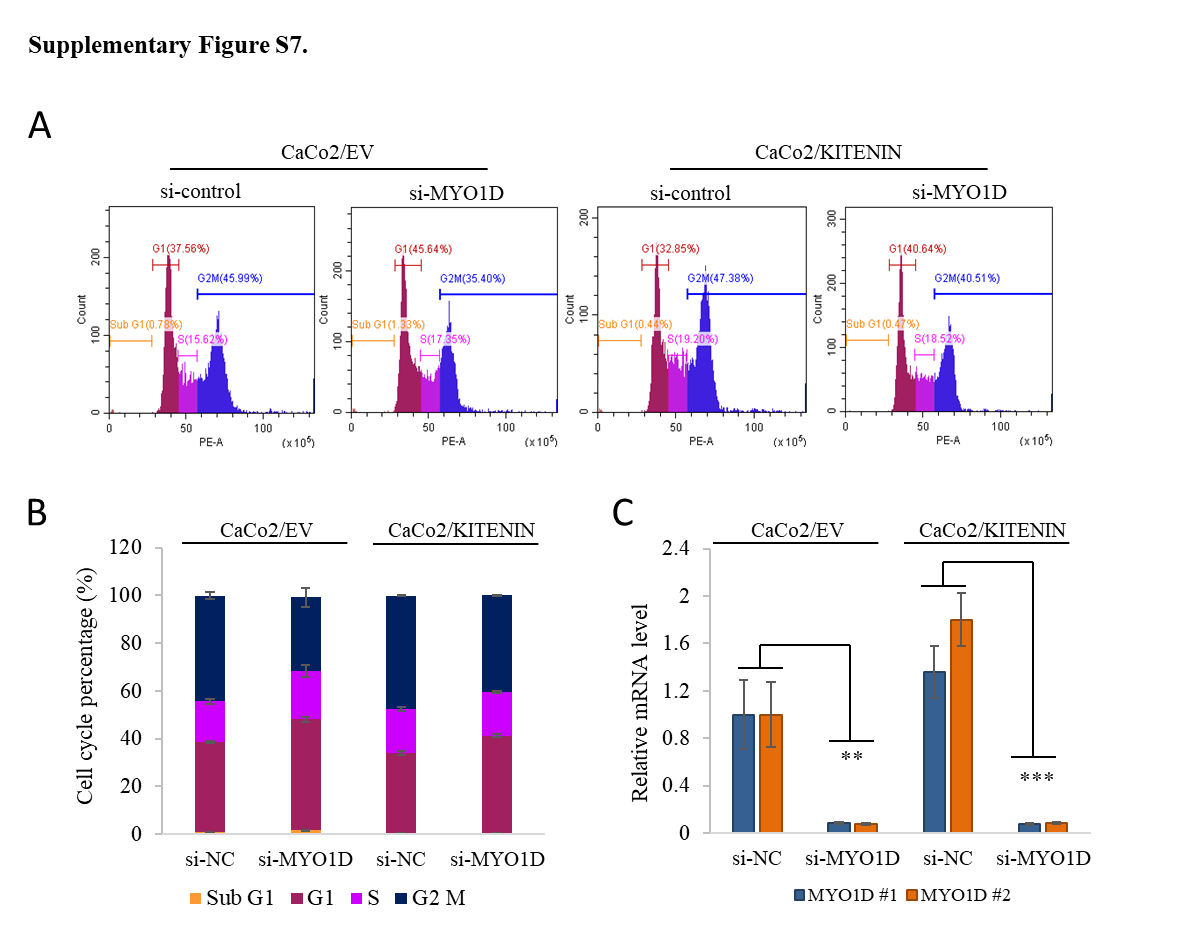
**

**
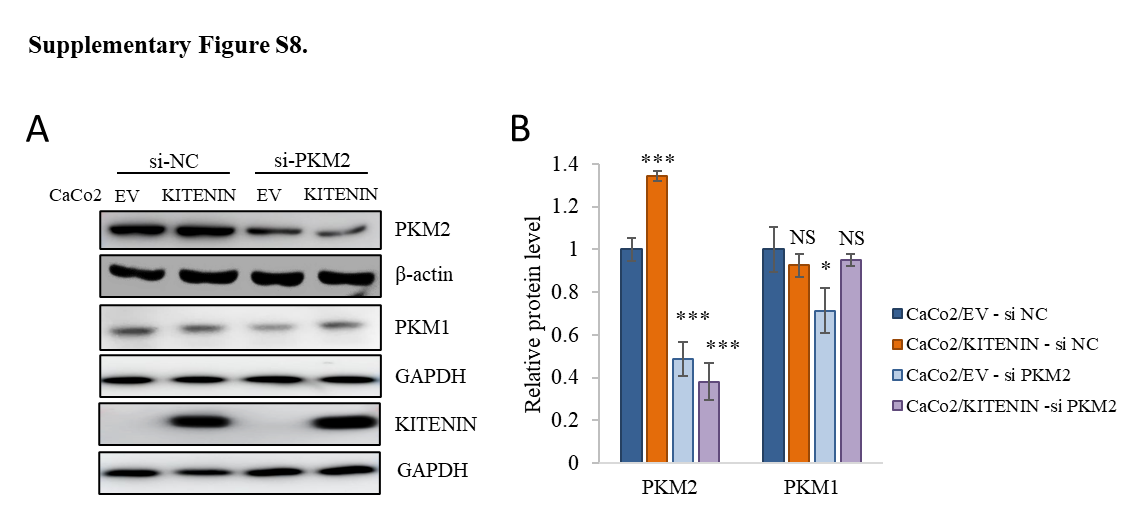
**

**
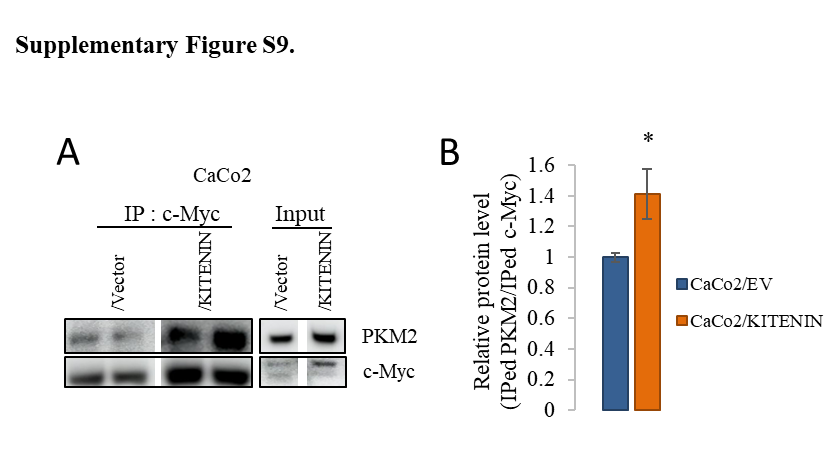
**

**
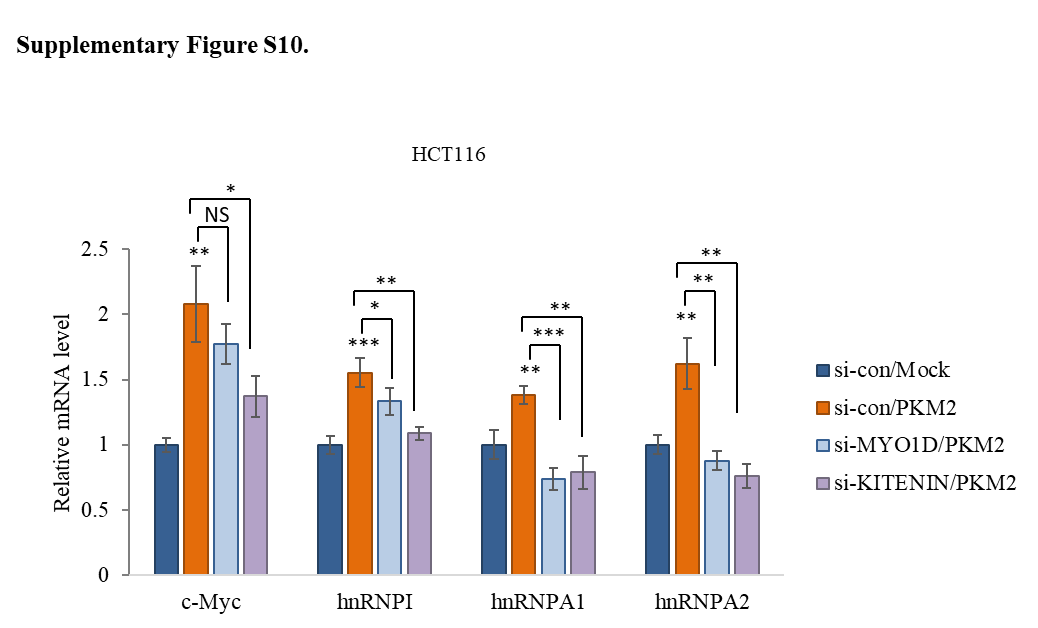
**

**
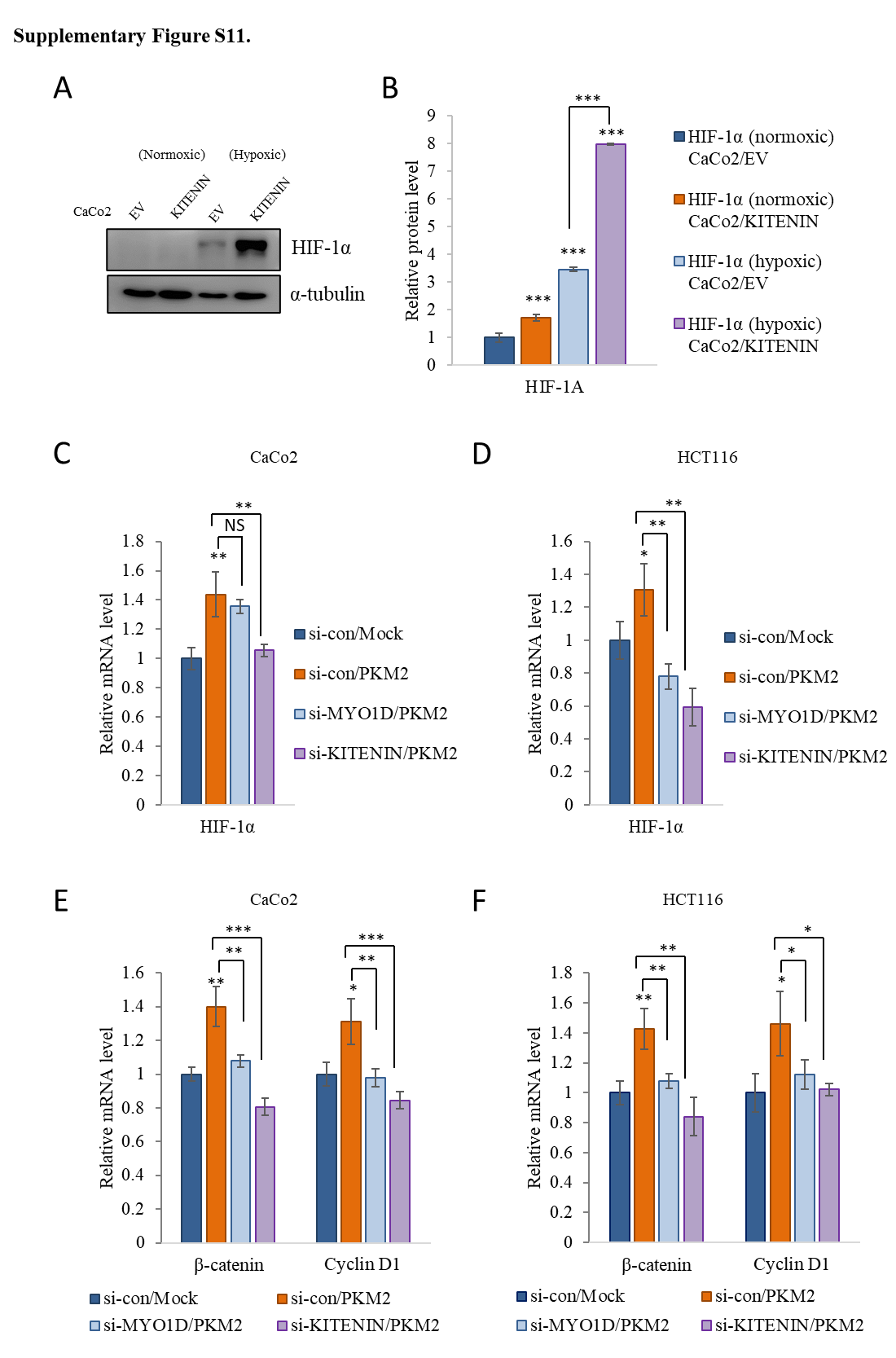
**

**
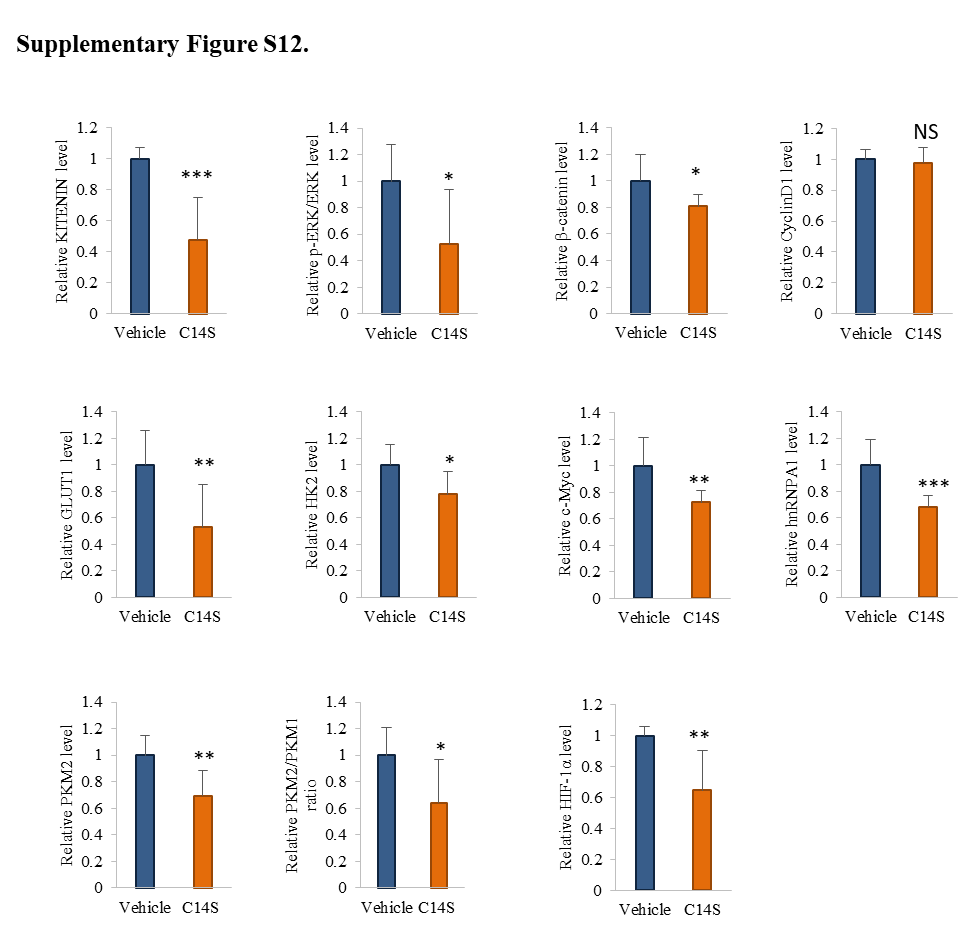
**

**Supplementary Tables**

**Supplementary Table S1.** Primer (Forward/Reverse) sequences.

| Gene symbol | Primers sequences | |
| --- | --- | --- |
|  | For (5'-3') | Rev (5'-3') |
| GLUT1 | CTTTGTGGCCTTCTTTGAAGT | CCACACAGTTGCTCCACAT |
| HK2 | AAGGCTTCAAGGCATCTG | CCACAGGTCATCATAGTTCC |
| PKM1 | CGAGCCTCAAGTCACTCCAC | GTGAGCAGACCTGCCAGACT |
| PKM2 | ATTATTTGAGGAACTCCGCCGCCT | ATTCCGGGTCACAGCAATGATGG |
| LDHA | TGGCCTGTGCCATCAGTATC | TTCCAAGCCACGTAGGTCAA |
| HIF-1α | ATCCATCTGACCATGAGGAAATG | TCGGCTAGTTAGGGTACACTTC |
| β-catenin | AAAATGGCAGTGCGTTTAG | TTTGAAGGCAGTCTGTCGTA |
| c-Myc | AATGAAAAGGCCCCCAAGGTAGTTATCC | GTCGTTTCCGCAACAAGTCCTCTTC |
| Cyclin D1 | CCGTCCATGCGGAAGATC | GAAGACCTCCTCCTCGCACT |
| CD44 | TGCCGCTTTGCAGGTGTAT | GGCCTCCGTCCGAGAGA |
| KITENIN | CGGAATAAAGACGGCAGAGG | TGCTCCGAGGTGCCTGTGAT |
| ErbB4 | ATGAAGCCGGCGACAGGACT | TTGCGCAAGGCTCGGTACTG |
| MYO1D#1 | TTTGCCTTCCGCCAGACATACG | CAGCCTCTTTGTCTGAAGGAAGG |
| MYO1D#2 | CACTTTTGTCCCTGTTGCTAA | ATTGCTCTGTCTTCCACCTTA |
| hnRNPI (PTBP1) | TCAGGCCTTCATCGAGATGCACA | TCTTGAGCTCCTTGTGGTTGGA |
| hnRNPA1 | GCTCACGGACTGTGTGGTAA | GGCCTTGCATTCATAGCTGC |
| hnRNPA2 | GGAGTGGAAGAGGAGGCAAC | CAGGTCCTCCTCCATACCCA |
| β-actin | ATTGTGAACTTTGGGGGATG | GATGAGATTGGCATGGCTTT |

**Supplementary Table S2.** Antibodies information

| **Antibody name** | **Product Information** |
| --- | --- |
| Beta-catenin antibody | Cell signaling, #9562 |
| Alpha tubulin (11h101) Rabbit mAb | Cell signaling, #2125 |
| GAPDH (D16H11) XP Rabbit mAb | Cell signaling, #5174 |
| Goat Anti-Rabbit IgG (H+L) Peroxidase Conjugated | Thermo scientific, 31460 |
| Goat Anti-Mouse IgG (H+L) Peroxidase Conjugated | Thermo scientific, NCI1430KR |
| PKM2 (D78A4) XP Rabbit mAB | Cell signaling, #4053 |
| GLUT1 (D3J3A) Rabbit mAB | Cell signaling, #12939 |
| HIF1A (D1S7W) XP Rabbit mAB | Cell signaling, #36169 |
| LDHA (C4B5) Rabbit mAB | Cell signaling, #3582 |
| Beta-actin antibody | Cell signaling, #4967 |
| Hexokinase II (C64G5) Rabbit mAb | Cell signaling, #2867 |
| Anti-cyclin D1 Ab-3 mouse mAB | Merck, Kenilworth, NJ, USA, DCS-6 |
| PKM1 (D30G6) Rabbit mAB | Cell signaling, #7067 |
| Anti-VANGL1 | Atlas antibodies, HPA025235 |
| Anti-Lamin B1 antibody – Nuclear Envelope Marker | Abcam, ab16048 |
| hnRNPA1 (D21H11) | Cell signaling, #8443 |
| hnRNPA2/B1 (2A2) Mouse mAb | Cell signaling, #9304 |
| CD44 (156-3C11) | Cell signaling, #3570 |
| c-Myc (9E10) | Santa Cruz Biotech., sc-40 |
| p44/42 MAPK (Erk1/2) (137F5) Rabbit mAb | Cell signaling, #4695 |
| Phospho-p44/42 MAPK (Erk1/2) (Thr202/Tyr204) Antibody | Cell signaling, #9101 |
| Goat Anti-Rabbit IgG (H+L) Peroxidase Conjugated | Thermo scientific, 31460 |
| Goat Anti-Mouse IgG (H+L) Peroxidase Conjugated | Thermo scientific, NCI1430KR |
